# Effects of Amino Acid Point Mutations on the Local Hydrophobicity, Structural Stability, and Conformational Flexibility of P-Glycoprotein

**DOI:** 10.3390/biom16071046

**Published:** 2026-07-17

**Authors:** Alexandra Ioana Năstasie, Adriana Isvoran

**Affiliations:** 1 Department of Biology, West University of Timișoara, 16 Pestalozzi, 300115 Timisoara, Romania; alexandra.nastasie@e-uvt.ro; 2 One Health Initiative-Focused Multidisciplinary Biosciences Advanced Research Center, West University of Timisoara, Oituz 4, 300086 Timisoara, Romania

**Keywords:** ABCB1, computational analysis, polymorphism

## Abstract

P-glycoprotein (P-gp, ABCB1) plays a central role in multidrug resistance and drug pharmacokinetics. In this study, an integrated computational analysis of point amino acid substitutions across the P-gp sequence was performed to evaluate their predicted pathogenicity and structural impact. Variant classification using AlphaMissense and PolyPhen-2 revealed concordant predictions for a substantial subset of amino acid substitutions, with 21 variants consistently classified as benign and 13 as pathogenic, while discrepancies for other variants reflected methodological differences between the tools. Notably, substitutions located in transmembrane domains were more frequently predicted to be deleterious compared with those in cytoplasmic or extracellular regions, consistent with the structural and functional constraints imposed on membrane-spanning helices. Changes in local hydropathicity, average flexibility, protein stability (ΔΔG), hydrogen-bonding patterns, surface hydrophobicity and electrostatic potential distributions were also evaluated. Stability predictions obtained using I-Mutant2.0 and DynaMut2.0 indicated that many substitutions tend to destabilize P-gp, although differences in ΔΔG values were observed between methods due to distinct algorithmic approaches. Structural superposition analyses demonstrated generally minor backbone deviations, yet local alterations in surface hydrophobicity, electrostatic potential, and hydrogen bond networks were evident. These physicochemical perturbations, even when subtle, may influence conformational dynamics and coupling between nucleotide-binding and transmembrane domains. Overall, these findings suggest that single amino acid substitutions may alter the local structural environment of P-gp and could potentially influence its transport function.

## 1. Introduction

P-glycoprotein (P-gp), also known as ATP-binding cassette subfamily B member 1 (ABCB1), is a membrane-bound protein encoded by the Multidrug Resistance 1 (MDR1) gene. It is expressed in various tissues involved in the absorption, distribution, metabolism, and excretion of xenobiotics, including the blood–brain barrier, intestine, liver, and kidney. P-gp functions as an ATP-driven efflux pump that exports chemically diverse drugs from cells [1]. This process reduces intracellular drug concentrations, significantly affecting drug absorption, distribution, and excretion, and its inhibition or induction can lead to drug–drug interactions. Its activity also plays a crucial role in pharmacokinetics and contributes to chemotherapy resistance by limiting the effectiveness of certain anticancer agents [2].

**Figure 1:**
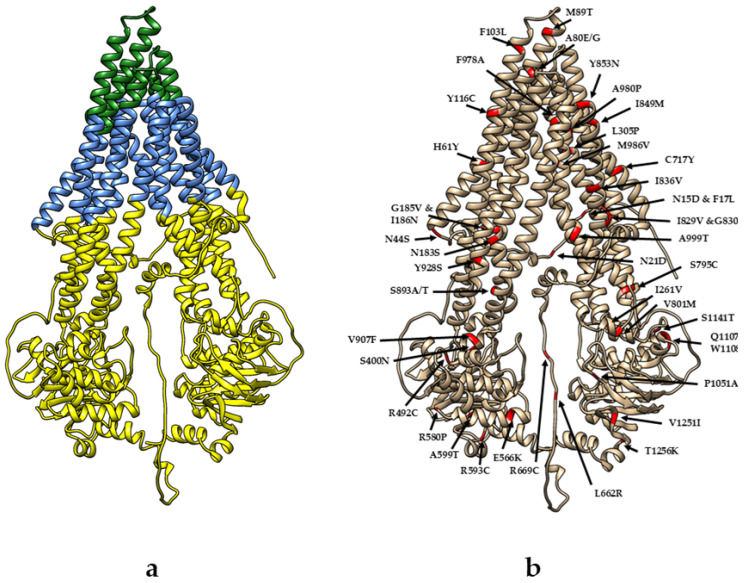
(**a**) Three-dimensional structure of P-glycoprotein, with the cytoplasmic domains (1–44, 138–186, 237–292, 353–711, 778–832, 875–934, and 996–1280) shown in yellow, the transmembrane domains (45–67, 117–137, 187–208, 216–236, 293–316, 331–352, 712–732, 757–777, 833–853, 855–874, 935–957, and 974–995) shown in blue, and the extracellular regions (68–116, 209–215, 317–330, 733–756, 854–855, and 958–973) shown in green; (**b**) schematic mapping of substitutions onto the P-gp structure.

Studies employing X-ray crystallography and sequence analysis reveal that P-gp is a protein which consists of four domains (Figure 1a): two transmembrane domains (TMD1 and TMD2), each containing six transmembrane helices (TMHs), and two cytoplasmic domains that are primarily constituted by the nucleotide-binding domains (NBD1 and NBD2), which are indispensable for providing the energy required for substrate transport through ATP binding and hydrolysis. TMD1 and NBD1 comprise the N-terminal half, homologous to the C-terminal half composed of TMD2 and NBD2. TMD1 and TMD2 create a large binding pocket, and NBD1 and NBD2 bind and hydrolyze ATP [3,4].

Literature data reveal that P-gp mediates efflux via mechanisms such as the hydrophobic vacuum cleaner and flippase models, thereby limiting cytotoxic and central nervous system drug effectiveness. These results clarify that P-gp’s structural complexity directly underpins its substrate promiscuity and its central role in multidrug resistance and barrier function [1].

Research has shown that both synonymous and nonsynonymous polymorphisms in P-gp can significantly alter its expression, function, and substrate specificity, affecting drug disposition across different ethnic populations resulting in an increased risk of toxicity [5]. These genetic variations can also affect protein conformation, stability, and recycling time, leading to changes in drug transport and response to inhibitors [6].

A literature search was conducted to identify single-point amino acid substitutions in P-gp sequence and to assess their reported effects on the protein’s structural features and function. Published studies have reported a total of 45 single-point amino acid substitutions in the P-gp sequence. These substitutions are mapped schematically onto the P-gp structure (Figure 1b), and their effects, when known, are summarized in Table 1.

Table 1 indicates that the variants are distributed across distinct regions of the protein structure: 31 substitutions (69%) occur in the cytoplasmic regions including NBD domains, 9 substitutions (20%) in the transmembrane domains, and 5 substitutions (11%) in the extracellular N-terminal domain. The table also highlights the limited understanding of how amino acid substitutions influence the structural characteristics and stability of P-gp. Polymorphisms resulting in amino acid substitutions at the cytoplasmic surface of P-gp have been reported to alter substrate specificity [13,14], whereas substitutions in the transmembrane regions may affect the conformational changes associated with drug transport [15]. Moreover, while the functional effects of several P-gp allelic variants have been extensively examined, many identified variants remain insufficiently characterized. Therefore, the objective of this study is to explore how single amino acid substitutions within the P-gp sequence impact the protein’s structural properties, stability, and flexibility in relation to its biological function, employing a computational approach.

## 2. Materials and Methods

To perform the computational study, the human P-glycoprotein sequence was retrieved from the UniProt database (ID: P08183) and manually modified to include the known amino acid substitutions. Although numerous structural files for P-glycoprotein are available in the Protein Data Bank (PDB), all contain unresolved regions. These missing segments, corresponding primarily to flexible loop and terminal regions, may affect the accuracy of structure-based predictions because they can play important roles in protein stability, dynamics, and interactions with ligands or substrates. Therefore, computational approaches that rely solely on available PDB structures may be limited in fully capturing the effects of amino-acid substitutions located in these regions. For this reason, the present study used the PDB structure with ID 7A65, as it contains a low number of missing residues, and structural models obtained using the SWISS-MODEL online tool (Swiss Institute of Bioinformatics, Basel, Switzerland) [16], which is continuously updated and provides a complete structural representation of the protein. Furthermore, in order to compare the local structural properties between the native and mutated protein, structural models of both wild-type and mutant P-gp were generated using the SWISS-MODEL after manually introducing the corresponding amino acid substitutions.

The AlphaMissense tool (version 2024) [17,18] was employed to assess the potential functional consequences of individual substitutions in P-glycoprotein by estimating the impact of each amino acid substitution on protein structure and function. This artificial intelligence-based method predicts whether a given missense variant is likely pathogenic or benign. AlphaMissense integrates structural information derived from AlphaFold models with evolutionary conservation data from humans and closely related primates to generate a pathogenicity score between 0 and 1 for every possible amino acid substitution, classifying variants as likely pathogenic (score ≥ 0.9), likely benign (score ≤ 0.34), or of uncertain significance (score between 0.35 and 0.89). This approach enables the identification substitutions most likely to disrupt P-gp folding, stability, or transport activity. For this analysis, the UniProt accession of human P-glycoprotein (P08183) was used, and all amino acid substitutions listed in Table 1 were evaluated.

Another computational approach employed to evaluate the effects of missense mutations on protein structure and function is Polymorphism Phenotyping version 2 (PolyPhen-2) [19]. PolyPhen-2 predicts the potential impact of amino acid substitutions by combining sequence conservation, physicochemical differences between residues, and structural context when experimental structures or homology models are available. PolyPhen-2 predicts a quantitative score between 0 and 1 reflecting the likelihood that a missense variant is damaging, which is subsequently used to classify the variant as benign (score ≤ 0.149), possibly damaging (score between 0.150 and 0.849), or probably damaging (score ≥ 0.850). The UniProt accession of human P-glycoprotein (P08183) was used as entry data for this analysis too, and all amino acid substitutions listed in Table 1 were evaluated.

Although numerous tools are available for predicting variant pathogenicity, AlphaMissense and PolyPhen-2 were selected because they rely on complementary computational frameworks and are among the most widely validated and commonly used methods for missense variant interpretation. The use of both tools enables cross-validation of predictions and increases confidence in variants with concordant classifications.

To obtain the hydropathicity and average flexibility profiles of P-gp, the ProtScale tool online tool was used [20]. ProtScale calculates position-specific property profiles, such as hydropathicity and average flexibility, from a protein’s amino-acid sequence using published amino-acid scales. Each scale assigns a numerical value to the amino acids according to a specific physicochemical or structural property such as hydropathicity, flexibility or secondary structure parameters. Hydropathicity describes the overall affinity of an amino acid or peptide region for polar versus nonpolar environments and integrates both hydrophobicity (water-repelling) and hydrophilicity (water-attracting) properties. The tool takes the protein sequence as input, allows the user to select the desired scale, and generates both numerical values and a profile plot showing how each property varies along the sequence. The output enables identification of sequence regions with distinct properties and supports comparative analyses in protein characterization studies. ProtScale can also be used to assess the effects of amino acid substitutions by comparing property profiles before and after substitution, thereby illustrating how a substitution alters local hydropathicity or flexibility at and around the mutated position. Such comparisons can reveal whether a substitution renders a region more hydrophobic or hydrophilic, or more flexible or rigid. In the present study, window sizes of 3, 5 and 9 amino acids were used (i.e., the number of neighboring residues averaged at each position), with the relative weight of the window edges set to 100% of the window center, and the scale left unnormalized (not scaled from 0 to 1).

The impact of single amino acid substitutions on protein structural stability was assessed using the I-Mutant2.0 [21] and DynaMut2.0 [22] computational tools. The combined use of these methods provides a more comprehensive assessment of substitutions-induced changes in protein stability. I-Mutant2.0 is a support vector machine-based tool designed to predict the effect of single amino acid substitutions on protein stability starting either from the protein sequence or structure if available. This method estimates the direction and magnitude of stability changes by computing the variation in Gibbs free energy (ΔΔG = ΔGmutant − ΔGwild-type) between the mutant and wild-type protein. I-Mutant2.0 can operate either with protein structural information or with sequence alone. It outputs both a predicted stability change (increase or decrease) and a quantitative ΔΔG value, reported in kcal/mol. The negative values of Gibbs free energy suggest a destabilizing effect on the protein’s structure, while the positive values suggest a stabilizing effect. DynaMut2.0 combines normal mode analysis with graph-based signatures representing the wild-type structural environment to understand how amino acid substitutions alter both stability and flexibility. It estimates changes in folding free energy (ΔΔG = ΔGmutant − ΔGwild-type) and helps to determine whether a substitution stabilizes (ΔΔG > 0) or destabilizes (ΔΔG < 0) the protein and highlights the regions whose mobility is affected. For analyses conducted using both I-Mutant2.0 and DynaMut2.0, the experimental structure with PDB ID 7A65 was used as the primary input. For residues not resolved in this structure, the corresponding Swiss models were employed to evaluate the effects of the associated substitutions. All the generated models have the Global Model Quality Estimation (GMQE) score of 0.85 suggesting that the generated homology models are expected to be of high reliability, although local structural uncertainties cannot be excluded. Although numerous tools are available for predicting the effects of amino-acid substitutions, I-Mutant2.0 and DynaMut2.0 were selected because they rely on complementary computational approaches and are widely validated.

Structural changes induced by amino acid substitutions were analyzed by visualizing and comparing protein structures using UCSF Chimera v1.19 [23]. Wild-type and mutant protein structures were imported into Chimera and superimposed to assess local conformational differences, as well as to compare hydrogen-bonding patterns, electrostatic potential distributions, and surface hydrophobicity between the native and mutant P-gp structures. This comparative visualization approach enabled the identification of regions in which substitutions induced local structural rearrangements or conformational changes.

**Figure 2:**
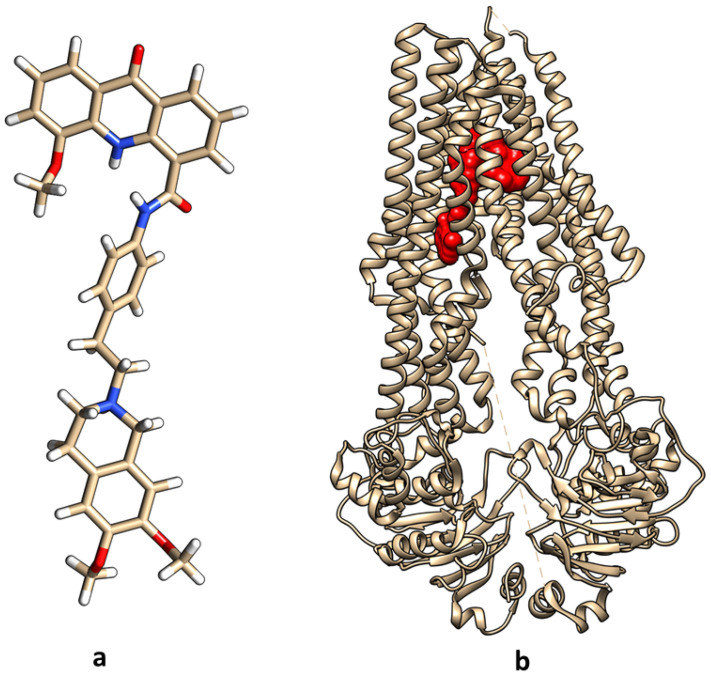
(**a**) Three-dimensional structure of elacridar represented as sticks and colored by atom type (oxygen, red; carbon, brown; nitrogen, blue; and hydrogen, white); (**b**) overall view of the P-gp conformation represented as a brown ribbon, showing the binding site of elacridar (red surface) within the drug-binding pocket.

To investigate substitution-induced conformational changes and their potential functional consequences, molecular docking was performed using the SwissDock 2024 server [24], followed by analysis of the predicted protein–ligand interactions using the Protein–Ligand Interaction Profiler (PLIP, version 2025) tool [25]. The SwissDock 2024 server is an online molecular docking platform that predicts binding modes and interactions between small molecules and target proteins. The docking calculations were performed using the AutoDock Vina algorithm (version 1.2.5) to explore possible ligand conformations and binding sites. The docking search space was graphically defined to encompass the binding pocket according to the ligand position observed in the cryo-EM structure with PDB ID 7A6C, corresponding to a complex of P-glycoprotein with the potent inhibitor elacridar [4]. The PDB ID 7A6C structure was selected because it contains two molecules of elacridar (Figure 2) allowing reliable identification of the binding pocket based on experimentally validated coordinates and thereby improving the docking setup. Structural studies indicate that elacridar occupies a binding site in P-glycoprotein located primarily within the transmembrane region, with interactions involving residues near the cytoplasmic interface of the transporter.

Analysis of structural file 7A6C reveals that 2 molecules of elacridar are bound in the central cavity of the P-glycoprotein. One molecule adopts a U-shaped conformation positioned entirely within the binding pocket, whereas the second molecule adopts an L-shaped conformation extending toward the cytoplasmic gate region of P-gp and displays weaker and less specific interactions with the protein [4]. The structure of the U-shaped elacridar–P-gp complex shows that elacridar binds through hydrophobic interactions with F336, F732, and F983, a hydrogen bond with Y953, and π-stacking interactions with F983. Based on the proximity of these residues to the elacridar binding pocket, mutant models carrying the substitutions L305P, C717Y, F978A, A980P, and M986V were generated in UCSF Chimera.

Each mutant structure was energy-minimized using 100 steps of steepest descent followed by 10 steps of conjugate gradient minimization prior to docking. To validate the docking protocol, elacridar was also docked into the native P-gp structure, and the predicted binding pose was compared with the experimentally determined binding mode.

PLIP is an automated computational tool used to identify and characterize non-covalent interactions between proteins and ligands in 3D structures. It detects interaction types such as hydrogen bonds, hydrophobic contacts, π-π stacking, salt bridges, and water-mediated interactions. It is widely used to analyze docking results to better understand binding modes and protein-ligand interaction patterns [25].

## 3. Results

Predictions of the potential deleterious effects of individual P-glycoprotein amino acid substitutions generated using PolyPhen-2 and AlphaMissense are presented in Table 2.

For many amino acid substitutions, the two tools provide concordant predictions, with 21 substitutions consistently classified as benign, 13 classified as pathogenic by both tools, and 3 classified as pathogenic by PolyPhen-2 but ambiguous by AlphaMissense. For the rest of P-glycoprotein alleles predictions obtained using the two tools are different, reflecting the distinct algorithms and training datasets used by each tool. While AlphaMissense incorporates large-scale deep mutational scanning data and structural context, PolyPhen-2 relies on evolutionary conservation and sequence-based features. As a result, the predicted impact of specific amino acid substitutions may vary between the two methods. Among the 24 amino acid substitutions located in the cytoplasmic regions with concordant predictions from the two tools, 16 are classified as benign and 7 as pathogenic. In the transmembrane regions, 7 amino acid substitutions showed concordant predictions, of which 2 were predicted to be benign and 5 to be pathogenic. In contrast, all three amino acid substitutions located in the extracellular region with consensus predictions were classified as benign. Overall, these results suggest that amino acid substitutions in the transmembrane regions of P-gp are more likely to be deleterious, consistent with previous observations reported for other membrane proteins [26]. The higher proportion of pathogenic predictions in transmembrane regions is in agreement with the structural and functional constraints imposed on these domains, which generally tolerate fewer amino acid substitutions. Furthermore, previous studies have shown that amino acid substitutions in transmembrane regions typically preserve residue non-polarity, whereas disease-associated variants more frequently introduce charged residues or involve unfavorable non-conservative substitutions, thereby disrupting membrane protein stability and function [27]. The computational prediction tools AlphaMissense and/or PolyPhen-2 have been widely applied and successfully validated the benign or pathogenic effects of point substitutions across various protein families [18,28].

**Figure 3:**
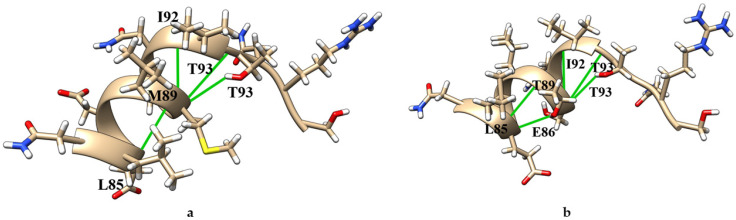
Comparison of the hydrogen-bonding interactions (green lines) of methionine (M) at position 89 in the native protein (**a**) and threonine (T) at position 89 in the polymorphic variant (**b**). The side-chain atoms are represented as sticks and colored according to atom type (oxygen, red; carbon, brown; nitrogen, blue; and hydrogen, white).

Changes in hydropathicity for the allelic variants calculated using sliding windows of 3, 5, and 9 amino acids are presented in the Supplementary Materials (Tables S1, S3 and S5), while the corresponding changes in average flexibility for these window sizes are provided in Tables S2, S4 and S6. Table 3 also summarizes the predicted effects of all amino acid substitutions on protein structural stability as determined using the I-Mutant2.0 and DynaMut2.0 computational tools. Predictions were primarily based on the experimental structure of P-gp (PDB ID 7A65). However, because this structure is incomplete, predictions for missing residues were generated using structural models obtained from the SWISS-MODEL online server. Changes in protein stability were expressed as variations in Gibbs free energy (ΔΔG) between the mutant and wild-type proteins, with negative ΔΔG values indicating destabilization and positive values indicating increased stability. Moreover, the number of hydrogen bonds formed by the native and mutated residues with neighboring amino acids was determined using Chimera 1.19 software, and corresponding data are presented in Table 3. Figure 3 illustrates the hydrogen-bonding interactions of methionine 89 in the native structure compared with those of threonine 89 in the polymorphic variant.

Data presented in Table 3 and Supplementary Tables S1–S6 generally reveal that changes in local hydropathicity and average flexibility associated with amino acid substitutions extend over a region spanning at least nine amino acids. Table 3 also reveals that, overall, both methods consistently identified substitutions that tend to compromise the structural stability of P-glycoprotein, irrespective of their location within the protein. Nevertheless, differences in the predicted ΔΔG values were observed between the two approaches. These discrepancies likely reflect the distinct methodological principles underlying each tool, as I-Mutant2.0 is based on machine learning-driven thermodynamic predictions, whereas DynaMut2.0 incorporates protein dynamics and conformational flexibility into its analyses. Accordingly, DynaMut2.0 may capture substitution-induced changes in protein flexibility that are not considered by I-Mutant2.0. The discrepancies observed among computational predictors highlight the limitations of relying on a single in silico approach for assessing the effects of amino acid substitutions.

**Figure 4:**
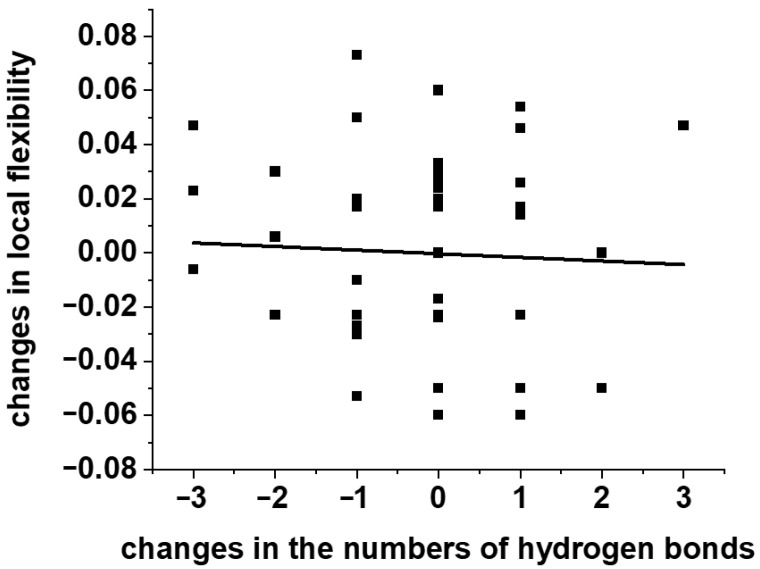
Relationship between changes in the number of hydrogen bonds and changes in local flexibility. The solid line indicates the linear regression fit, showing a weak negative correlation between the two variables, suggesting that increases in hydrogen bond number are only slightly associated with decreased local flexibility (Pearson correlation coefficient, r = −0.05).

**Figure 5:**
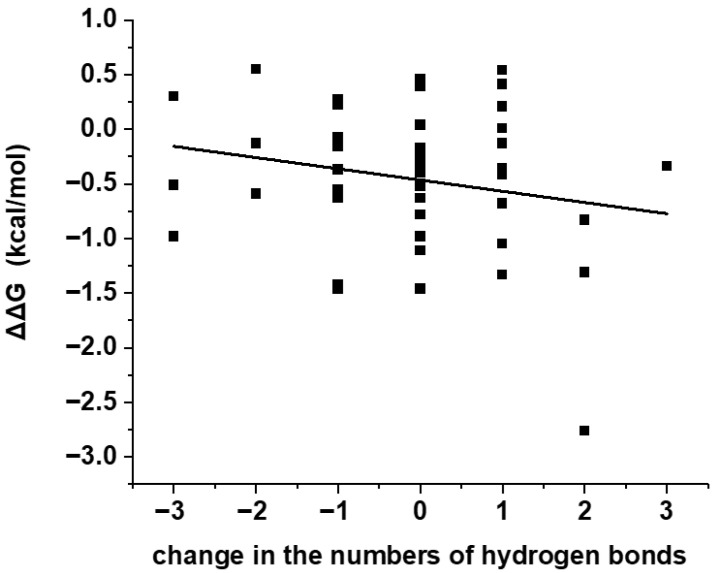
Plot of ΔG (kcal/mol) values predicted by DynaMut2.0 based on Swiss-model structures versus change in the number of hydrogen bonds. The regression line indicates a weak negative correlation between the two variables (Pearson correlation coefficientt, r = −0.21).

To address the relationship between physicochemical changes and structural stability in a quantitative manner, correlation analyses were performed and visualized using scatter plots for all point amino acid substitutions. Specifically, relationships between changes in local flexibility, ΔΔG values predicted by DynaMut2.0 based on Swiss-model structures, and differences in hydrogen bond counts between native and mutant residues were evaluated. The resulting plots (Figure 4 and Figure 5) reveal the extent to which local physicochemical perturbations are associated with changes in protein stability and dynamic behavior. Pearson correlation coefficients were calculated to support these observations; the results are reported in the figure legends.

**Figure 6:**
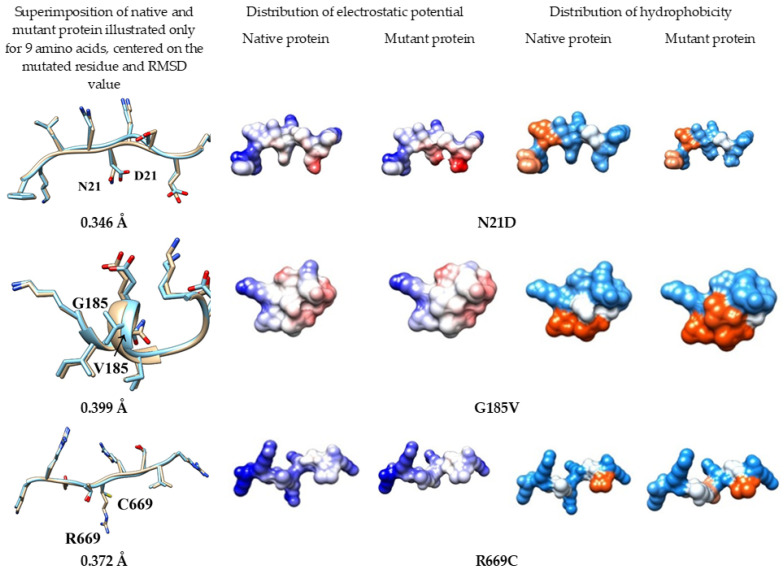
Overlay of the native and mutant protein structures highlighting a nine-residue region centered on the substituted amino acid (native protein—brown; mutant protein—blue), together with a comparison of the local electrostatic potential and surface hydrophobicity distributions for the P-glycoprotein variants exhibiting the highest RMSD values. Hydrophobic regions are shown in orange, whereas hydrophilic regions are shown in blue. In the electrostatic potential mapping, negatively charged regions are colored red and positively charged regions are colored blue. The RMSD values (0.346 Å, 0.399 Å, and 0.372 Å) represent the root-mean-square deviation between the atomic coordinates of the native and mutant structures following structural superimposition of the nine-residue region surrounding the amino acid substitution.

Figure 6 illustrates the superimposed native and mutant protein structures for a nine-residue region centered on the substituted amino acid, together with the distributions of electrostatic potential and surface hydrophobicity, for the P-glycoprotein variants exhibiting the highest RMSD values. The complete set of allelic variants is presented in Supplementary Table S7.

Figure 6 and Supplementary Table S7 show that the RMSD values obtained from the structural superimposition of the native and mutant proteins are generally low, indicating the absence of significant backbone deviations in the analyzed regions and suggesting only minor local structural adjustments. In addition, Figure 6 and Supplementary Table S7 reveal changes in the distribution of local surface hydrophobicity and electrostatic potential in regions of P-gp harboring amino acid substitutions compared with the wild-type protein. These findings are consistent with the data presented in Table 1 and Table 3.

**Figure 7:**
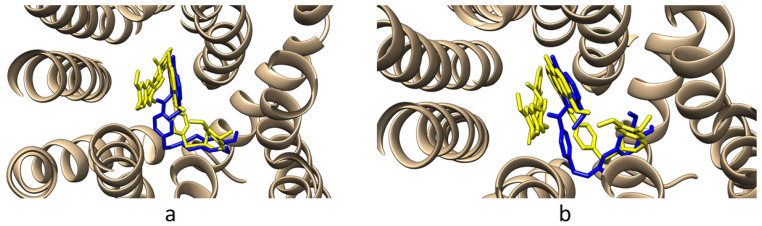
Best-docked pose of elacridar (blue sticks) superimposed on the U-shaped and L-shaped conformations of elacridar observed in the cryo-EM structure (PDB ID: 7A6C; yellow sticks) for the native P-glycoprotein (**a**) and the F978A mutant (**b**).

The results of the molecular docking analysis are presented in Figure 7, illustrating the predicted interactions of native P-gp (Figure 7a) and its F978A variant (Figure 7b) with elacridar.

Figure 7a shows that the best-docked pose of elacridar in the native protein corresponds to its U-shaped conformation observed in the cryo-EM structure, demonstrating that the docking protocol successfully reproduce the key protein–ligand interactions. Furthermore, molecular docking results indicate that the best binding poses of elacridar correspond to the position of the U-shaped ligand within the binding pocket. As a representative example, the F978A variant is shown in Figure 7b.

Table 4 summarizes the predicted binding free energies of elacridar with the native protein and its mutant variants, together with the protein–ligand interactions identified using the PLIP tool. For comparison, the table also includes the protein–ligand interactions between molecule 1 of elacridar and P-gp, observed in the cryo-EM structure.

The docking scores and PLIP interaction analysis suggested that elacridar adopts a broadly similar binding mode in the native P-gp structure and the investigated mutant variants (Table 4). The predicted binding free energy of the mutants differed only marginally from that of the native protein, with score differences ranging from 0.069 to 0.587 kcal/mol. Given the inherent uncertainty of docking scoring functions, these differences should not be interpreted as evidence of meaningful changes in binding affinity. Rather, the results suggest that the investigated substitutions are unlikely to substantially alter the predicted binding mode of elacridar.

Compared with the cryo-EM structure, docking of the native model predicted an expanded hydrophobic interaction network involving residues L65, F72, F336, I340, F343, F728, and F983, which was associated with a favorable predicted binding free energy (ΔG = −10.985 kcal/mol). The conservation of the aromatic residues F336, F728, and F983 among most variants suggests that these residues may contribute to the predicted binding of elacridar. Among the analyzed mutants, L305P exhibited the most favorable predicted binding free energy (ΔG = −11.060 kcal/mol), although the difference relative to the native protein was small and should be interpreted cautiously given the inherent limitations of docking scoring functions. This variant also displayed an additional hydrophobic contact with E875, which may reflect a local rearrangement of the binding site and could contribute to the predicted binding pose. Likewise, the persistence of the Y953 hydrogen bond and F983 π-stacking interaction across all complexes suggests that these interactions may play an important role in the predicted recognition of elacridar by P-gp.

## 4. Discussion

The present work integrates pathogenicity prediction, structural-stability evaluation, hydropathicity profiling, flexibility analysis, and molecular docking simulations to assess how naturally occurring amino acid substitutions may influence the function of P-gp. Collectively, the results indicate that although many variants are likely to be benign, several (particularly those located within transmembrane segments) could significantly affect protein stability, conformational behavior, or ligand interactions.

Pathogenicity predictions from PolyPhen-2 and AlphaMissense were largely consistent, identifying 21 variants as benign and 13 as deleterious. However, the two tools disagreed for several substitutions, which is expected given their different methodological approaches. PolyPhen-2 relies heavily on evolutionary conservation and physicochemical features, whereas AlphaMissense uses deep-learning models trained on extensive mutational and structural datasets. Previous studies have shown that evolutionary conservation-based tools frequently overestimate the pathogenicity of substitutions occurring at highly conserved positions, whereas deep learning approaches may better capture structural tolerance and compensatory effects within protein folds [29]. Accordingly, the benign predictions generated by AlphaMissense for the Y116C, R492C, R593C, A599T, S795C, and Q1107P variants suggest that these substitutions may be better tolerated than indicated by PolyPhen-2, although their functional impact requires experimental validation.

If only amino acid substitutions predicted as damaging/pathogenic or benign by both PolyPhen-2 and AlphaMissense are considered, variants predicted to be pathogenic are approximately 2.5–3 times more likely to exhibit a decrease in local hydropathicity than variants predicted to be benign. In contrast, no clear association is observed between predicted pathogenicity and changes in local flexibility, as pathogenic and benign variants display similar frequencies of increased and decreased flexibility. These observations suggest that, within this dataset, alterations in local hydropathicity may be a better indicator of deleterious P-glycoprotein amino acid substitutions than changes in local flexibility.

Correlation analysis of Table 3 indicated that changes in local hydropathicity and flexibility are weakly associated with predicted stability changes (ΔΔG) across all computational approaches. Hydropathicity exhibited weak to moderate positive Pearson correlations with ΔΔG (r = 0.22 for I-Mutant2.0, r = 0.38 for DynaMut2.0, and r = 0.44 for DynaMut2.0 Swiss-model), suggesting that alterations in the local hydrophobic environment contribute to protein stability perturbations, with structure-based predictors capturing this relationship more effectively than sequence-based methods. In contrast, flexibility changes showed weak negative correlations with ΔΔG (r = −0.32, −0.17, and −0.20 for I-Mutant2.0, DynaMut2.0, and Swiss-model, respectively), indicating a modest tendency for increased local flexibility to be associated with destabilizing effects. Overall, these results suggest that hydropathicity exerts a comparatively more consistent influence on ΔΔG than flexibility; however, the generally low correlation magnitudes highlight that additional structural determinants beyond local physicochemical properties likely govern stability changes in P-glycoprotein.

Furthermore, changes in hydrogen bonding showed only a negligible association with local structural flexibility (Figure 4). Specifically, the number of hydrogen bonds exhibited an extremely weak negative correlation with flexibility (Pearson’s r = −0.05), and the linear regression fit indicated no meaningful predictive relationship. This suggests that variations in hydrogen bond counts alone are insufficient to explain changes in local mobility, implying that protein flexibility is governed by a broader set of structural and energetic determinants rather than hydrogen bonding in isolation. Similarly, when examining the relationship between hydrogen bond changes and predicted stability, a weak negative correlation was observed between ΔG values (as predicted by DynaMut2.0 based on Swiss models) and hydrogen bond variation (Figure 5; r = −0.21). Although this trend suggests a slight tendency for increased hydrogen bonding to be associated with marginally more favorable stability changes, the magnitude of the correlation indicates that hydrogen bonds account for only a limited proportion of the observed variability in ΔG. Overall, these results indicate that while hydrogen bonding may contribute modestly to stability changes, it is not the principal determinant of either local flexibility or global stability within the studied system.

Many substitutions altered local physicochemical profiles across several neighboring residues, indicating that even single-site substitutions may influence the local structural environment beyond the substitution site. Because P-glycoprotein is a membrane protein, changes in hydropathicity may be particularly relevant: increased hydropathicity could favor membrane compatibility, packing, or folding, whereas decreased hydropathicity may reduce these properties and increase solvent exposure. Several variants identified as deleterious in the present analysis involve substantial physicochemical changes that may affect local structure or membrane compatibility. Likewise, substitutions predicted to increase local flexibility may alter conformational behavior, whereas those predicted to decrease flexibility could restrict local structural rearrangements. Although these predictions do not directly demonstrate altered protein dynamics, they suggest that certain amino acid substitutions may have functional consequences even when they do not occur within a known active site, by modifying the local physicochemical and structural environment of the protein.

The results of the present study regarding the effects of amino acid substitutions on the local physicochemical properties, structural characteristics, and predicted function of P-glycoprotein are generally consistent with published data, where available (Table 1). Alterations in P-gp function have the potential to influence the transport of drugs and other substrates across cell membranes, thereby affecting drug accumulation, multidrug resistance, toxicity, or pharmacokinetics. Several variants predicted to be deleterious in the present study have previously been reported to alter the transport of specific substrates, resulting in either reduced or enhanced transport activity, which provides additional support for the computational predictions. Predicted changes in protein stability are also consistent with previous observations that amino acid substitutions can modify local residue interactions, the surrounding structural environment, and protein flexibility, potentially influencing the overall structural integrity of the protein [22,30].

Although the overall trends predicted by I-Mutant2.0 and DynaMut2.0 were largely consistent, differences in ΔΔG values were observed for several variants. These discrepancies likely arise from the distinct methodological frameworks employed by the two approaches. Whereas I-Mutant2.0 primarily estimates thermodynamic stability changes based on machine learning-based models, DynaMut2.0 additionally incorporates information on protein dynamics and conformational flexibility. Because P-glycoprotein undergoes substantial conformational changes during its transport cycle, predictions that account for structural flexibility may provide complementary information regarding the potential effects of amino acid substitutions. Therefore, combining multiple computational approaches provides a more comprehensive assessment of the possible structural consequences of P-gp missense variants than reliance on a single predictive method.

However, experimental structural and functional data are available for only a limited subset of the analyzed variants, including N21D, G185V, I186N, L305P, S400N, C717Y, G830V, S893A, S893T, V907F, and F978A (Table 1). Among the variants analyzed, N21D, G185V, and R669C exhibited the largest local conformational differences, as reflected by the RMSD values shown in Figure 6.

However, experimental structural and functional data are available for only a limited subset of the analyzed variants, including N21D, G185V, I186N, L305P, S400N, C717Y, G830V, S893A, S893T, V907F, and F978A (Table 1). Among the variants analyzed, N21D, G185V, and R669C exhibited the largest local conformational differences, as reflected by the RMSD values shown in Figure 6. These predictions are consistent with published studies reporting that the N21D substitution alters local folding and residue interactions [5,9], whereas the G185V substitution induces increased rigidity within the transmembrane domain [12]. Likewise, the predicted deleterious effect of G185V is in agreement with experimental evidence showing that substitutions at residue G185 can alter substrate specificity and impair P-glycoprotein transport activity [12].

To the best of our knowledge, no experimental evidence is currently available regarding the structural effects of the R669C substitution. The present analysis suggests that this variant may influence the local structural organization of P-gp. I186N substitution replaces a hydrophobic isoleucine with a polar asparagine and was consistently predicted by all methods to have a strongly destabilizing effect on protein stability. This prediction is consistent with experimental studies showing that the I186N substitution alters drug-induced conformational transitions and modifies substrate interactions in P-gp, supporting the hypothesis that this substitution perturbs the local structural environment [13]. Several substitutions, including L305P, R580P, C717Y, V907F, A980P have previously reported to reduce substrates transport by P-gp [8]. Among these, the proline substitutions L305P, and A980P were predicted to produce strongly destabilizing effects (ΔΔG = −2.38 kcal/mol for L305P and ΔΔG = −2.63 kcal/mol for A980P), accompanied by increases in local flexibility despite maintaining or increasing the number of hydrogen bonds. Furthermore, the F971–P996 segment forms part of the substrate-binding pocket [4], highlighting the potential structural and functional relevance of the A980P substitution. The Y928S variant was also predicted to have a pronounced destabilizing effect (ΔΔG = −3.01 kcal/mol). Because tyrosine residues frequently participate in hydrogen bonding and π-stacking interactions with ligands or neighboring helices [4], substitution with the smaller serine residue may alter the local hydropathicity and increase local flexibility, potentially affecting the structural environment of the substrate-binding pocket. The R580P substitution markedly reduced the predicted number of hydrogen bonds (from four to one), suggesting disruption of the local electrostatic interaction network. Previous studies have also shown that the S400N substitution may alter the conformation of NBD1 and thereby influence the transport cycle [9], whereas molecular dynamics simulations have suggested that G830V induces reorganization of the substrate-binding cavity [12]. Finally, the W1108R substitution replaces a bulky aromatic residue with a positively charged arginine and was predicted to alter the local physicochemical environment. Although the structural effects of this substitution have not been experimentally investigated, charged substitutions within transmembrane helices have been reported to destabilize membrane proteins [31], suggesting that W1108R may have similar structural consequences.

Result from the present study are generally consistent with published data. A previous investigation combining experimental characterization of the kinetic properties of polymorphic P-glycoprotein variants with computational analyses reported that substitutions such as S400N, R492C, R669C, I849M, S893A, T893A, M986V, A999T, and P1051A produced moderate changes in the kinetic properties of P-glycoprotein and affected substrate specificity to a greater extent than overall protein stability. The same study suggested that A999T and P1051A altered local flexibility, whereas S893A altered hydrogen bonding and T893A modified local flexibility [32]. Because the structural and functional consequences of many of the investigated variants remain unknown, the present results provide complementary predictions regarding both their pathogenicity and their potential structural effects.

In addition to local conformational changes, point amino acid substitutions were predicted to alter the number of hydrogen bonds, suggesting modifications in the local intramolecular interaction network. The M89T variant provides a representative example, where replacement of methionine with threonine reduced hydrogen bonding from four interactions to one. Such changes may influence local structural stability even in the absence of substantial backbone rearrangements. Substitutions such as L305P, I186N, Y928S, and N15D were predicted to increase the hydrogen bonds formed by the substituted residue, which may partially offset destabilizing effects associated with altered local packing. Conversely, variants such as Y116C, C717Y, and W1108R were predicted to reduce the number of hydrogen bonds and may therefore modify the local interaction network. These observations suggest that the structural consequences of amino acid substitution depend not only on changes in physicochemical properties but also on their influence on the surrounding interaction environment. Nevertheless, RMSD analysis revealed generally modest structural deviations between the native and mutant models, indicating that most substitutions are predicted to induce local rather than global conformational changes. This observation is in line with previous studies suggesting that many pathogenic missense mutations may subtly alter protein dynamics or interaction networks without necessarily causing complete structural collapse. Increased hydrogen bonding within the TMD regions may stabilize helix packing and alter the geometry of the drug-binding cavity, potentially influencing substrate affinity and specificity [33]. Although enhanced hydrogen bonding could improve thermodynamic stability, excessive rigidity may limit the dynamic flexibility required for efficient substrate extrusion. Conversely, reduced hydrogen bond interactions may increase structural flexibility but potentially at the expense of stability. In the TMDs, decreased hydrogen bonding could weaken helix–helix interactions, possibly leading to broadening or distortion of the drug-binding cavity. Such changes may impair substrate recognition or affect the coupling between ATP hydrolysis and substrate translocation. Overall, these findings suggest that hydrogen bond networks contribute to both the structural integrity and functional dynamics of P-glycoprotein. Alterations in either direction—increased or decreased hydrogen bonding—may disturb the delicate balance between stability and flexibility required for efficient transport.

Similar effects have been reported for other proteins. Single amino acid substitutions have been shown to alter local conformation, surface hydrophobicity, and flexibility, with structural perturbations extending beyond the substitution site. In some cases, such changes have been associated with reduced protein stability, altered active-site dynamics, or decreased substrate affinity, as reported for family 1 sulfotransferases [34,35], lysozyme [36], the SRC SH3 domain, and the serine proteinase inhibitor CI-2 [37]. These observations are consistent with the present findings, suggesting that local structural perturbations induced by amino acid substitutions may have broader functional consequences even when the overall protein fold is largely preserved.

The findings of this study based on molecular docking analyses, suggest that the analyzed P-gp amino acid substitutions exert only limited effects on the predicted interactions with the inhibitor. Although minor differences in hydrophobic contacts and docking scores were observed, the conservation of key hydrogen-bonding and aromatic interactions indicates that the overall predicted binding mode is largely retained among the investigated variants. Experimental studies will be required to determine whether these subtle differences translate into changes in inhibitor potency or efficacy.

Given the difficulty and cost of experimentally characterizing every variant, computational predictions provide a valuable means of prioritizing substitutions that are most likely to affect function for subsequent experimental investigation. For P-gp, such predictions may contribute to a better understanding of the drug resistance in cancer and other diseases and help guide future functional studies.

## 5. Conclusions

This study shows that single amino acid substitutions in P-glycoprotein are predicted to influence structural stability, hydrogen bonding, hydropathicity, flexibility, and the distribution of electrostatic potential. Although many variants were associated with only minor backbone deviations, they may still modify the local physicochemical environment of the protein and thereby influence the conformational behavior and interdomain communication involved in ATP-driven substrate transport. The predicted changes in hydrogen-bonding networks and local stability further suggest that maintaining an appropriate balance between structural rigidity and flexibility may be important for transporter function. The differences observed between computational predictors highlight the value of integrative approaches that combine evolutionary, structural, and dynamic information. Given the limited availability of experimental structural data for many variants, the computational framework presented here provides a bases for prioritizing substitutions for future functional validation. These findings improve our understanding of the potential structural consequences of naturally occurring amino acid substitutions in P-glycoprotein and may contribute to future studies of drug resistance, pharmacokinetics, and personalized therapeutic strategies. However, because all analyses were performed in silico, experimental validation using transport assays, ATPase activity measurements, and biophysical stability studies will be necessary to confirm the predicted functional effects of the identified variants.

## Figures and Tables

**Table 1:** Single-point amino acid substitutions in the P-gp sequence identified from the published literature and their reported effects on P-gp structure and/or function.

No.	Amino Acid Substitution	Domain	Sub-Region	Known Effects of Amino Acid Substitutions on P-gp Structure and/or Function
1	N15D [7]	Cytoplasmic N-terminal domain		unknown effect
2	F17L [7]	Cytoplasmic N-terminal domain		unknown effects
3	N21D [5]	Cytoplasmic N-terminal domain		This substitution selectively decreases Calcein-AM transport while largely sparing BODIPY-FL-paclitaxel and digoxin transport [5]. Furthermore, it seems to enhance transport of N-methyl-quinidine, but does not affect the transport of aliskiren [8]. The N21D substitution adds a negative charge in a cytoplasmic loop before TM1, likely altering local folding and NBD interactions, which may impact P-gp conformation and ATPase activity [5,9].
4	N44S [7]	Cytoplasmic N-terminal domain		Unknown effects
5	H61Y [8]	Helical TMD1	TM1	This substitution does not affect the transport of N-methyl-quinidine but slightly increases the transport of aliskiren [8].
6	A80E [6]	Extracellular domain	ECL1	Unknown effects
7	A80G [7]	Extracellular domain	ECL1	Unknown effects
8	M89T [10]	Extracellular domain	ECL1	Increased P-gp function, leading to higher resistance to multiple drugs like daunorubicin, doxorubicin, actinomycin D or valinomycin [10].
9	F103L [11]	Extracellular domain	ECL1	Unknown effects
10	Y116C [8]	Extracellular domain	ECL1	This substitution does not affect the transport of N-methyl-quinidine and aliskiren [8].
11	N183S [7]	Intracellular domain	ICL1	This substitution does not affect the transport of N-methyl-quinidine and aliskiren [8].
12	G185V [6]	Intracellular domain	ICL1	The rigidification of the TMH3 region causes structural rearrangement of the transmembrane domain, leading to substrate-specific changes in P-gp activity, enhancing resistance to certain drugs while reducing it for others [12].
13	I186N [13]	Intracellular domain	ICL1	The substitution increases TMH3 polarity and enhances P-gp drug transport and resistance [13].
14	I261V [7]	Intracellular domain	ICL2	This substitution does not affect the transport of N-methyl-quinidine and aliskiren [8].
15	L305P [8]	Helical TMD1	TM5	Decreased transport activity to about 25% of wild-type P-gp for N-methyl-quinidine and aliskiren. L305 is located in TM5 and its substitution with a proline residue introduces a rigid hinge that may disrupts the protein helical structure and integrity explaining the observed reduction in the activity [8].
16	S400N [5]	NBD1		This change can modify ATP binding and hydrolysis, reducing substrate transport affinity and resulting in increased resistance to drugs such as doxorubicin, vinblastine, and vincristine, along with decreased absorptive transport across epithelial barriers. Substitution of Ser400 with asparagine may alter NBD1, as the slightly bulkier side chain of asparagine could affect ATP binding or hydrolysis and thereby influence drug translocation cycles [9].
17	R492C [7]	NBD1		Unknown effects
18	E566K [6]	NBD1		Unknown effects
19	R580P [8].	NBD1		Decreased N-methyl-quinidine and aliskiren transport activity to approximately 40–50% of wild-type P-gp, indicating partial loss of function [8].
20	R593C [7]	NBD1		Unknown effects
21	A599T [7]	NBD1		Unknown effects
22	L662R [10]	Intracellular domain	ICL3	Increased transport of doxorubicin and vaninomycin, leading to higher drug resistance. No significant effect on the transport of actinomycin D or daunorubicin [10].
23	R669C [10]	Intracellular domain	ICL3	This substitution reduces calcein-AM transport, increases actinomycin D and daunorubicin transport, enhancing resistance to these drugs while having no significant effect on BODIPY-FL-paclitaxel, doxorubicin, or valinomycin transport [10].
24	C717Y [8]	Helical TMD2	TM7	P-gp exhibited complete loss of function for aliskiren and N-methylquinidine. C717, located in TM7 with its side chain facing TM8, when replaced by tyrosine, may perturb the TM7–TM8 interface and compromise transporter function [8].
25	S795C [8]	Intracellular domain	ICL4	Reduced N-methyl-quinidine transport activity, indicating partial loss of function, while aliskiren transport is not significantly affected, showing a substrate-dependent functional effect [8].
26	V801M [7]	Intracellular domain	ICL4	Unknown effects
27	I829V [7]	Intracellular domain	ICL4	Unknown effects
28	G830V [12]	Intracellular domain	ICL4	Reorientation of TMH9 and TMD [12].
29	I836V [7]	Helical TMD2	TM9	This substitution does not affect the transport of N-methyl-quinidine and aliskiren [8].
30	I849M [7]	Helical TMD2	TM9	Unknown effects
31	Y853N [8]	Helical TMD2	TM9	No significant change in aliskiren transport, while the transport of N-methyl-quinidine transport was decreased [8].
32	S893A [8]	Intracellular domain	ICL5	The substitution enhances drug efflux, reducing responsiveness to chemotherapy with Fluorouracil, Adriamycin and Cytoxan, while Ser or Thr at this position is linked to better drug response [9]. This substitution does not affect the transport of N-methyl-quinidine and aliskiren [8]. The S893 is located in the cytoplasmic region of TM10 in the internal tunnel inner hinge domain between the NBD. TheS893A change may alter TM10 dynamics and hinge movement, affecting P-gp conformational changes [9].
33	S893T [8]	Intracellular domain	ICL5	This substitution does not affect the transport of N-methyl-quinidine and aliskiren [8]. The S893T substitution may influence ATP binding and stabilization [9].
34	V907F [8]	NBD2		Decreased transport activity to less than 25% of wild-type P-gp for both N-methyl-quinidine and aliskiren. V907 is located in intracellular loop 4 between TM10 and TM11 of P-gp. The inward-facing side chain lies within a tight turn between two helices, and replacement of valine with bulky phenylalanine may perturb the local conformation [8].
35	Y928S [8]	NBD2		Aliskiren transport unaffected, while N-methyl-quinidine transport reduced; meaning that function is substrate-specific [8].
36	F978A [12]	Helical TMD2	TM12	Affects the rearrangement of transmembrane helices in the C-terminal half [12].
37	A980P [8]	Helical TMD2	TM12	Decreased N-methyl-quinidine and aliskiren transport activity to approximately 40–50% of wild-type P-gp, showing partial loss of function [8].
38	M986V [7]	Helical TMD2	TM12	Unknown effects
39	A999T [7]	NBD2 Cytoplasmic C-terminal domain		Unknown effects
40	P1051A [10]	NBD2 Cytoplasmic C-terminal domain		Unknown effects
41	Q1107P [7]	NBD2 Cytoplasmic C-terminal domain		Unknown effects
42	W1108R [10]	NBD2 Cytoplasmic C-terminal domain		Unknown effects
43	S1141T [10]	NBD2 Cytoplasmic C-terminal domain		Unknown effects
44	V1251I [5]	NBD2 Cytoplasmic C-terminal domain		Unknown effects
45	T1256K [7]	NBD2 Cytoplasmic C-terminal domain		Unknown effects

ICL—intracellular loop, ECL—extracellular loop.

**Table 2:** Predicted deleterious effects of individual P-glycoprotein amino acid substitutions assessed using the PolyPhen-2 and AlphaMissense computational tools.

Amino Acid Substitution	Sub-Region	PolyPhen-2	AlphaMissense
N15D		Benign	Benign
F17L		Benign	Benign
N21D		Benign	Benign
N44S		Benign	Benign
H61Y	TM1	Probably damaging	Likely pathogenic
A80E	ECL1	Possibly damaging	Likely pathogenic
A80G	ECL1	Benign	Benign
M89T	ECL1	Benign	Benign
F103L	ECL1	Benign	Benign
Y116C	ECL1	Probably damaging	Benign
N183S	ICL1	Possibly damaging	Benign
G185V	ICL1	Probably damaging	Likely pathogenic
I186N	ICL1	Probably damaging	Likely pathogenic
I261V	ICL2	Benign	Benign
L305P	TM5	Probably damaging	Likely pathogenic
S400N		Benign	Benign
R492C		Probably damaging	Benign
E566K		Probably damaging	Likely pathogenic
R580P		Probably damaging	Likely pathogenic
R593C		Probably damaging	Benign
A599T		Probably damaging	Benign
L662R	ICL3	Benign	Benign
R669C	ICL3	Benign	Benign
C717Y	TM7	Probably damaging	Likely pathogenic
S795C	ICL4	Probably damaging	Benign
V801M	ICL4	Benign	Benign
I829V	ICL4	Benign	Benign
G830V	ICL4	Probably damaging	Likely pathogenic
I836V	TM9	Benign	Benign
I849M	TM9	Probably damaging	Ambiguous
Y853N	TM9	Probably damaging	Ambiguous
S893A	ICL5	Benign	Benign
S893T	ICL5	Benign	Benign
V907F		Probably damaging	Likely pathogenic
Y928S		Probably damaging	Ambiguous
F978A	TM12	Probably damaging	Likely pathogenic
A980P	TM12	Probably damaging	Likely pathogenic
M986V	TM12	Benign	Benign
A999T		Benign	Benign
P1051A		Benign	Benign
Q1107P		Probably damaging	Benign
W1108R		Probably damaging	Likely pathogenic
S1141T		Benign	Benign
V1251I		Benign	Benign
T1256K		Probably damaging	Benign

Green cells indicate substitutions predicted to be benign, orange cells indicate ambiguous predictions, and red cells indicate substitutions predicted to be pathogenic. White cells correspond to substitutions for which the two computational tools yielded discordant predictions.

**Table 3:** Changes in local hydropathicity and average flexibility caused by amino acid substitutions.

Amino Acid Substitution	Sub-Region	Effect on Local Hydropathicity	Effect on Local Flexibility	ΔΔG (kcal/mol)			H Bonds Made by Native Residue	H Bonds Made by Mutated Residue
				I-Mutant2.0 PDB ID 7A65	DynaMut2.0 for PDB ID 7A65	DynaMut2.0 Swiss Models		
N15D		0	0.017	/	/	0.210	0	1
F17L		0.330	0.020	/	/	−0.360	0	0
N21D		0	0.017	/	/	0.040	0	0
N44S		0.900	0.017	−1.590	0.050	0.220	3	2
H61Y	TM1	0.633	0.033	−0.490	0.250	0.390	3	3
A80E	ECL1	−1.766	0.046	−2.170	−0.090	−1.330	2	3
A80G	ECL1	−0.733	0.060	−2.540	−0.070	−1.460	2	2
M89T	ECL1	−0.867	0.047	/	/	0.300	4	1
F103L	ECL1	0.333	0.020	/	/	−0.370	1	0
Y116C	ECL1	1.267	−0.023	0.850	0.660	0.550	2	0
N183S	ICL1	0.900	0.017	−1.190	0.260	0.010	1	2
G185V	ICL1	1.534	−0.050	−3.670	−1.240	−0.830	1	3
I186N	ICL1	−2.667	0.000	−2.230	−2.520	−2.760	1	3
I261V	ICL2	−0.100	−0.023	−0.400	−1.260	−1.110	2	2
L305P	TM5	−1.800	0.047	−2.380	0.300	−0.340	2	5
S400N		−0.900	−0.017	−0.500	−0.520	−0.630	1	1
R492C		2.333	−0.060	−0.600	0.740	−0.170	3	3
E566K		−0.133	−0.010	−1.810	−0.400	−0.630	2	1
R580P		0.967	−0.006	−1.930	−0.480	−0.510	4	1
R593C		2.333	−0.060	−0.370	0.750	0.540	0	1
A599T		−0.833	0.027	−2.450	−0.750	−0.780	2	2
L662R	ICL3	−2.767	0.054	/	/	0.410	0	1
R669C	ICL3	2.333	−0.060	/	/	0.460	0	0
C717Y	TM7	−1.267	0.023	0.080	−1.260	−0.980	3	0
S795C	ICL4	1.100	−0.053	−1.830	−0.410	−0.160	4	3
V801M	ICL4	−0.767	−0.030	−0.880	−0.010	0.270	1	0
I829V	ICL4	−0.100	−0.023	−0.730	−1.570	−1.050	1	2
G830V	ICL4	1.533	−0.050	−1.510	1.210	−0.130	2	3
I836V	TM9	−0.100	−0.023	−0.770	−0.010	−0.590	2	0
I849M	TM9	−0.867	−0.053	−1.120	0.090	−0.070	2	1
Y853N	TM9	−0.733	0.014	−2.160	−0.010	−0.360	1	2
S893A	ICL5	0.867	−0.050	−1.050	−0.530	−0.420	2	3
S893T	ICL5	0.033	−0.024	−1.580	−0.670	−0.400	2	2
V907F		−0.467	−0.027	−1.480	−0.980	−1.420	3	2
Y928S		0.167	0.030	−3.010	−1.740	−1.310	2	4
F978A	TM12	−0.334	0.017	−2.130	−1.780	−0.980	2	2
A980P	TM12	−1.134	0.050	−2.630	0.150	−0.100	2	1
M986V	TM12	0.767	0.030	−0.680	−1.470	−0.520	2	2
A999T		−0.833	0.026	−1.160	−0.380	−0.680	2	3
P1051A		1.133	−0.050	−1.680	0.150	−0.330	0	0
Q1107P		0.633	0.006	−1.310	−0.150	−0.130	3	1
W1108R		−1.200	0.073	−2.430	−0.330	−1.460	3	2
S1141T		0.033	−0.023	0.150	−0.280	−0.550	2	1
V1251I		0.100	0.024	−0.770	−0.450	−0.500	0	0
T1256K		−1.067	0.000	−0.310	−0.060	−0.250	3	3

Expressed as Δ values (mutant − native) and calculated using a three-residue sliding window. The table also presents the predicted effects of each amino acid substitution on the structural stability of P-glycoprotein, as determined using the I-Mutant2.0 and DynaMut2.0 computational tools, together with the number of hydrogen bonds formed by the native and mutant residues. Green cells indicate no change, orange cells indicate increases, and blue cells indicate decreases in local hydropathicity and average flexibility. Red cells indicate destabilizing effects, whereas yellow cells indicate stabilizing effects. Residues missing from the experimental structure are shown in white.

**Table 4:** Predicted binding free energies and protein–elacridar interactions for the native P-gp and its mutant variants, as identified using the PLIP tool. For comparison, the protein–ligand interactions observed in the cryo-EM structure are also included. The number of interactions is given in parentheses, where applicable.

Structure	ΔG (kcal/mol)	Hydrophobic Interactions	Hydrogen Bonds	Π-Stacking
Cryo-EM structure	-	F336, F732, F983	Y953	F983
native	−10.985	L65, F72, F336, I340 (2), F343, F728, F983	Y950 Y953	F983
L305P	−11.060	F72, F336, I340, F343 (2), F728, E 75, F 83 (3)	Y953	F983
C717Y	−10.412	L65, F336, I340, F343, F728, F983 (2)	Y953	F983
F978A	−10.398	L65, F336, I340, F343 (2), F728, F983 (2)	Y953	F983
A980P	−10.569	L65, F336, I340, F343, F728, F983 (2)	Y953	F983
M986V	−10.916	L65, F336, F728, F983 (2)	Y953	F983

## Data Availability

The original contributions presented in this study are included in the article/Supplementary Materials. Further inquiries can be directed to the corresponding author(s).

## Supplementary Materials

The following supporting information can be downloaded at: https://www.mdpi.com/article/10.3390/biom16071046/s1, Table S1: Changes in local hydropathicity resulting from amino acid substitutions. Hydropathicity values were calculated using the ProtScale tool with a three-residue sliding window. The window centered on the substituted residue is highlighted in light orange for the wild-type protein and in blue for the mutant proteins. Regions unaffected by the substitutions are omitted; Table S2: Changes in local flexibility resulting from amino acid substitutions. Flexibility values were calculated using the ProtScale tool with a three-residue sliding window. The window centered on the substituted residue is highlighted in light orange for the wild-type protein and in blue for the mutant proteins. Regions unaffected by the substitutions are omitted; Table S3: Changes in local hydropathicity resulting from amino acid substitutions. Hydropathicity values were calculated using the ProtScale tool with a five-residue sliding window. The window centered on the substituted residue is highlighted in light orange for the wild-type protein and in blue for the mutant proteins. Regions unaffected by the substitutions are omitted; Table S4: Changes in local flexibility resulting from amino acid substitutions. Flexibility values were calculated using the ProtScale tool with a five-residue sliding window. The window centered on the substituted residue is highlighted in light orange for the wild-type protein and in blue for the mutant proteins. Regions unaffected by the substitutions are omitted; Table S5: Changes in local hydropathicity resulting from amino acid substitutions. Hydropathicity values were calculated using the ProtScale tool with a nine-residue sliding window. The window centered on the substituted residue is highlighted in light orange for the wild-type protein and in blue for the mutant proteins. Regions unaffected by the substitutions are omitted; Table S6: Changes in local flexibility resulting from amino acid substitutions. Flexibility values were calculated using the ProtScale tool with a nine-residue sliding window. The window centered on the substituted residue is highlighted in light orange for the wild-type protein and in blue for the mutant proteins. Regions unaffected by the substitutions are omitted; Table S7: Superimposition of the native and mutant protein structures highlighting a nine-residue region centered on the substituted amino acid (native protein, brown; mutant protein, blue), together with a comparison of the local electrostatic potential and surface hydrophobicity distributions between native and mutant P-glycoprotein. Hydrophobic regions are shown in orange, whereas hydrophilic regions are shown in blue. In the electrostatic potential maps, negatively charged regions are colored red and positively charged regions are colored blue.

## Abbreviations

The following abbreviations are used in this manuscript:

P-gp	P-glycoprotein
MDR1	Multidrug Resistance 1
TMD	transmembrane domain
TMHs	transmembrane helices
NBD	nucleotide-binding domain
PolyPhen-2	Polymorphism Phenotyping v2
