# Supplementary material for: Effects of Amino Acid Point Mutations on the Local Hydrophobicity, Structural Stability, and Conformational Flexibility of P-Glycoprotein
Source: Biomolecules. 2026 Jul 17;16(7):1046. doi: 10.3390/biom16071046 (PMC13406932; doi:10.3390/biom16071046)
Supplement: Supplementary file 1 [file biomolecules-16-01046-s001.zip › biomolecules-4275607-supplementary.pdf]

Effects of amino acid point mutations on the local hydrophobicity, structural stability, and conformational flexibility of P-glycoprotein

Alexandra Ioana Năstasie[1, 2], Adriana Isvoran[1, 2*]

[1]Department of Biology, West University of Timisoara, 16 Pestalozzi, 300115, Romania
[2]One Health Initiative-Focused Multidisciplinary Biosciences Advanced Research Center, West University of Timisoara, Oituz 4, 300086 Timisoara, Romania, alexandra.nastasie@e-uvt.ro, adriana.isvoran@e-uvt.ro
*Correspondence: adriana.isvoran@e-uvt.ro; tel: +40 (745) 901 850

Supplementary material

Table S1. Changes in local hydropathicity resulting from amino acid substitutions. Hydropathicity values were calculated using the ProtScale tool with a three-residue sliding window. The window centered on the substituted residue is highlighted in light orange for the wild-type protein and in blue for the mutant proteins. Regions unaffected by the substitutions are omitted.

Aminoacid number and mutation	Hidropathicity values for 3 aminoacids windows for wild-type protein	Hidropathicity values for allelic sequences with mutations: N15D, N21D, N44S, H61Y, A80G, M89T, F103L, Y116C, N183S, I186N, I261V, L305P, S400N, R492C, E566K, R580P, R593C, A599T, L662R, R669C, C717Y, V801M, , I829V, I836V, I849M, Y853N, S893A, V907F, Y928S, F978A, M986V, A999T, P1051A, Q1107P, S1141T, V1251I	Hidropathicity values for allelic sequences with mutations: F17L, A80E, G185V, G830V, S893T, A980P, W1108R, T1256K
In the region 2-12 there are not changes			
13	-3.900	-3.900	-3.900
14	-3.767	-3.767	-3.767
N15D	-1.533	-1.533	-1.533
16	0.700	0.700	1.033
F17L	0.567	0.567	0.900
18	0.900	0.900	1.233
19	-1.200	-1.200	-1.200
20	-1.067	-1.067	-1.067
N21D	-3.633	-3.633	-3.633

22	-2.733	-2.733	-2.733
23	-2.733	-2.733	-2.733
		In the region 24-41 there are not changes	
42	-2.200	-2.200	-2.200
43	-1.867	-0.967	-1.867
N44S	-1.733	-0.833	-1.733
45	-0.200	0.700	-0.200
46	-0.200	-0.200	-0.200
		In the region 47-58 there are not changes	
59	3.600	3.600	3.600
60	1.933	2.567	1.933
H61Y	0.300	0.933	0.300
62	-0.600	0.033	-0.600
63	0.333	0.333	0.333
		In the region 64-77 there are not changes	
78	1.267	1.267	1.267
79	3.033	2.300	1.267
A80G / A80E	0.367	-0.367	-1.400
81	0.033	-0.700	-1.733
82	-0.700	-0.700	-0.700
83	-0.700	-0.700	-0.700
84	-0.033	-0.033	-0.033
85	-1.067	-1.067	-1.067
86	-1.067	-1.067	-1.067
87	-1.067	-1.067	-1.067
88	0.733	-0.133	0.733
M89T	1.633	0.767	1.633
90	-0.800	-1.667	-0.800
91	0.067	0.067	0.067

In the region 92-100 there are not changes			
101	-1.533	-1.533	-1.533
102	0.567	0.900	0.567
F103L	1.733	2.067	1.733
104	2.500	2.833	2.500
105	0.400	0.400	0.400
In the region106-113 there are not changes			
114	-1.333	-1.333	-1.333
115	-0.267	1.000	-0.267
Y116C	-0.267	1.000	-0.267
117	-1.300	-0.033	-1.300
118	-1.133	-1.133	-1.133
In the region 119-180 there are not changes			
181	-0.067	-0.067	-0.067
182	-0.967	-0.067	-0.967
N183S	-0.833	0.067	-0.833
184	-2.467	-1.567	-0.933
G185V	0.200	-2.467	1.733
I186N	1.233	-1.433	2.767
187	0.200	-2.467	0.200
188	-2.600	-2.600	-2.600
In the region 189-258 there are not changes			
259	2.467	2.467	2.467
260	2.700	2.600	2.700
I261V	0.600	0.500	0.600
262	-0.233	-0.333	-0.233
263	-0.333	-0.333	-0.333
In the region 264-302 there are not changes			
303	2.800	2.800	2.800
304	3.467	1.667	3.467

L305P	4.033	2.233	4.033
306	2.333	0.533	2.333
307	1.667	1.667	1.667
In the region 308-397 there are not changes			
398	1.267	1.267	1.267
399	-0.400	-1.300	-0.400
400	0.233	-0.667	0.233
401	-1.233	-2.133	-1.233
402	-1.233	-1.233	-1.233
In the region 403-489 there are not changes			
490	-2.067	-2.067	-2.067
491	-2.067	0.267	-2.067
R492C	-2.800	-0.467	-2.800
493	-3.833	-1.500	-3.833
494	-0.933	-0.933	-0.933
564	-1.667	-1.667	-1.667
565	-2.600	-2.733	-2.600
E566K	-0.833	-0.967	-0.833
567	0.833	0.700	0.833
568	3.400	3.400	3.400
In the region 569-577 there are not changes			
578	-2.933	-2.933	-2.933
579	-2.933	-1.967	-2.933
R580P	-1.867	-0.900	-1.867
581	-1.967	-1.000	-1.967
582	1.033	1.033	1.033
In the region 583-590 there are not changes			
591	0.900	0.900	0.900
592	-0.333	2.000	-0.333
R593C	-1.267	1.067	-1.267

594	-2.067	0.267	-2.067
595	-1.733	-1.733	-1.733
596	0.833	0.833	0.833
597	1.733	1.733	1.733
598	3.500	2.667	3.500
A599T	1.967	1.133	1.967
600	1.400	0.567	1.400
601	-0.367	-0.367	-0.367
		In the region 602-659 there are not changes	
660	-2.033	-2.033	-2.033
661	0.733	-2.033	0.733
L662R	2.500	-0.267	2.500
663	1.267	-1.500	1.267
664	-1.300	-1.300	-1.300
665	-4.300	-4.300	-4.300
666	-3.067	-3.067	-3.067
667	-2.000	-2.000	-2.000
668	-2.000	0.333	-2.000
R669C	-3.233	-0.900	-3.233
670	-3.267	-0.933	-3.267
671	-0.367	-0.367	-0.367
		In the region 672-714 there are not changes	
715	2.200	2.200	2.200
716	3.167	1.900	3.167
C717Y	2.367	1.100	2.367
718	2.933	1.667	2.933
719	3.600	3.600	3.600
		In the region 720-792 there are not changes	
793	0.833	0.833	0.833
794	-0.833	0.267	-0.833

S795C	-1.133	-0.033	-1.133
796	1.633	2.733	1.633
797	0.400	0.400	0.400
798	-1.400	-1.400	-1.400
799	-3.833	-3.833	-3.833
800	-0.933	-1.700	-0.933
V801M	-0.033	-0.800	-0.033
802	0.833	0.067	0.833
803	0.367	0.367	0.367
In the region 804-826 there are not changes			
827	-0.833	-0.833	-0.833
828	1.967	1.867	1.967
I829V	1.967	1.867	3.500
G830V	1.100	1.000	2.633
831	-1.900	-0.367	-0.367
832	-0.500	-0.500	-0.500
833	0.367	0.367	0.367
834	3.267	3.267	3.267
835	3.500	3.400	3.500
I836V	2.667	2.567	2.667
837	0.100	0.000	0.100
838	-2.567	-2.567	-2.567
In the region 839-846 there are not changes			
847	2.867	2.867	2.867
848	4.500	3.633	4.500
I849M	2.733	1.867	2.733
850	2.167	1.300	2.167
851	2.167	2.167	2.167
852	2.000	1.267	2.000
Y853N	0.933	0.200	0.933

854	-0.867	-1.600	-0.867
855	-1.600	-1.600	-1.600
In the region 856-890 there are not changes			
891	-0.033	-0.033	-0.033
892	-1.567	-0.700	-1.533
S893A / S893T	-0.533	0.333	-0.500
894	-1.700	-0.833	-1.667
895	0.067	0.067	0.067
In the region 896-904 there are not changes			
905	-0.800	-0.800	-0.800
906	-0.333	-0.800	-0.333
V907F	2.567	2.100	2.567
908	2.533	2.067	2.533
909	2.400	2.400	2.400
In the region 910-925 there are not changes			
926	-0.300	-0.300	-0.300
927	0.433	0.600	0.433
Y928S	-2.467	-2.300	-2.467
929	-3.100	-2.933	-3.100
930	-2.933	-2.933	-2.933
In the region 931-975 there are not changes			
976	3.933	3.933	3.933
977	3.600	3.267	3.600
F978A	2.067	1.733	2.067
979	1.267	0.933	0.133
A980P	1.733	1.733	0.600
981	3.400	3.400	2.267
982	3.733	3.733	3.733
983	2.200	2.200	2.200

984	1.400	1.400	1.400
985	1.100	1.867	1.100
M986V	1.833	2.600	1.833
987	2.633	3.400	2.633
988	1.867	1.867	1.867
In the region 989-996 there are not changes			
997	-2.133	-2.133	-2.133
998	-1.000	-1.833	-1.000
A999T	-1.133	-1.967	-1.133
1000	-0.100	-0.933	-0.100
1001	-2.000	-2.000	-2.000
In the region 1002-1048 there are not changes			
1049	-0.200	-0.200	-0.200
1050	-0.200	0.933	-0.200
P1051A	2.367	3.500	2.367
1052	2.133	3.267	2.133
1053	1.500	1.500	1.500
In the region 1154-1104 there are not changes			
1105	1.500	1.500	1.500
1106	-0.933	-0.300	-0.933
Q1107P	-0.067	0.567	-1.267
W1108R	-0.200	0.433	-1.400
1109	-0.533	-0.533	-1.733
1110	0.367	0.367	0.367
In the region 1111-1138 there are not changes			
1139	1.300	1.300	1.300
1140	2.533	2.567	2.533
S1141T	-0.033	0.000	-0.033
1142	-2.600	-2.567	-2.600
1143	-3.500	-3.500	-3.500

In the region 1144-1248 there are not changes			
1249	-2.800	-2.800	-2.800
1250	-0.233	-0.133	-0.233
W1251I	-1.400	-1.300	-1.400
1252	-1.067	-0.967	-1.067
1253	-3.533	-3.533	-3.533
1254	-2.367	-2.367	-2.367
1255	-1.433	-2.500	-1.433
T1256K	-1.433	-2.500	-1.433
1257	-2.467	-3.533	-2.467
1258	-3.400	-3.400	-3.400
In the region 1259-1279 there are not changes			

Table S2. Changes in local flexibility resulting from amino acid substitutions. Flexibility values were calculated using the ProtScale tool with a three-residue sliding window. The window centered on the substituted residue is highlighted in light orange for the wild-type protein and in blue for the mutant proteins. Regions unaffected by the substitutions are omitted.

Aminoacid number and mutation	Flexibility values for 3 aminoacids windows for wild-type protein	Flexibility values for allelic sequences with mutations: N15D, N21D, N44S, H61Y, A80G, M89T, F103L, Y116C, N183S, I186N, I261V, L305P, S400N, R492C, E566K, R580P, R593C, A599T, L662R, R669C, C717Y, V801M, I829V, I836V, I849M, Y853N, S893A V907F, Y928S, F978A, M986V, A999T, P1051A, Q1107P, S1141T, V1251I, T1256K	Flexibility values for allelic sequences with mutations: F17L, A80E, G185V, G830V, S893T, A980P, W1108R
In the region 2-12 there are no changes			
13	0.470	0.470	0.470
14	0.467	0.483	0.467
N15D	0.413	0.430	0.413
16	0.360	0.377	0.380
F17L	0.363	0.363	0.383
18	0.383	0.383	0.403
19	0.433	0.433	0.433
20	0.430	0.447	0.430
N21D	0.463	0.480	0.463
22	0.480	0.497	0.480
23	0.493	0.493	0.493
In the region 24-41 there are no changes			
42	0.487	0.487	0.487
43	0.463	0.480	0.463
N44S	0.427	0.443	0.427
45	0.380	0.397	0.380
46	0.397	0.397	0.397
In the region 47-58 there are no changes			
59	0.427	0.427	0.427
60	0.413	0.447	0.413
H61Y	0.440	0.473	0.440
62	0.407	0.440	0.407

63	0.480	0.480	0.480
In the region 64-77 there are no changes			
78	0.427	0.427	0.427
79	0.377	0.437	0.423
A80G/ A80E	0.377	0.437	0.423
81	0.393	0.453	0.440
82	0.453	0.453	0.453
In the region 83-86 there are no changes			
87	0.460	0.460	0.460
88	0.393	0.440	0.393
M89T	0.393	0.440	0.393
90	0.423	0.470	0.423
91	0.477	0.477	0.477
In the region 92-100 there are no changes			
101	0.497	0.497	0.497
102	0.430	0.450	0.430
F103L	0.387	0.407	0.387
104	0.307	0.327	0.307
105	0.357	0.357	0.357
In the region 106-113 there are no changes			
114	0.437	0.437	0.437
115	0.400	0.377	0.400
Y116C	0.400	0.377	0.400
117	0.420	0.397	0.420
118	0.450	0.450	0.450
In the region 119-180 there are no changes			
181	0.480	0.480	0.480
182	0.463	0.480	0.463
N183S	0.473	0.490	0.473

184	0.500	0.517	0.450
G185V	0.500	0.500	0.450
I186N	0.513	0.513	0.463
187	0.503	0.503	0.503
188	0.507	0.507	0.507
In the region 189-258 there are no changes			
259	0.363	0.363	0.363
260	0.393	0.370	0.393
I261V	0.450	0.427	0.450
262	0.477	0.453	0.477
263	0.453	0.453	0.453
In the region 264-302 there are no changes			
303	0.347	0.347	0.347
304	0.350	0.397	0.350
L305P	0.400	0.447	0.400
306	0.417	0.463	0.417
307	0.413	0.413	0.413
In the region 308-397 there are no changes			
398	0.340	0.340	0.340
399	0.380	0.363	0.380
S400N	0.413	0.397	0.413
401	0.480	0.463	0.480
402	0.480	0.480	0.480
In the region 403-489 there are no changes			
490	0.497	0.497	0.497
491	0.497	0.437	0.497
R492C	0.523	0.463	0.523
493	0.497	0.437	0.497
494	0.450	0.450	0.450
In the region 495-563 there are no changes			

564	0.483	0.483	0.483
565	0.503	0.493	0.503
E566K	0.457	0.447	0.457
567	0.417	0.407	0.417
568	0.380	0.380	0.380
In the region 569-577 there are no changes			
578	0.513	0.513	0.513
579	0.513	0.507	0.513
R580P	0.503	0.497	0.503
581	0.470	0.463	0.470
582	0.447	0.447	0.447
In the region 583-590 there are no changes			
591	0.447	0.447	0.447
592	0.453	0.393	0.453
R593C	0.460	0.400	0.460
594	0.450	0.390	0.450
595	0.443	0.443	0.443
596	0.420	0.420	0.420
597	0.453	0.453	0.453
598	0.403	0.430	0.403
A599T	0.453	0.480	0.453
600	0.403	0.430	0.403
601	0.453	0.453	0.453
In the region 602-659 there are no changes			
660	0.517	0.517	0.517
661	0.463	0.517	0.463
L662R	0.447	0.500	0.447
663	0.453	0.507	0.453
664	0.487	0.487	0.487
In the region 665-666 there are no changes			

667	0.493	0.493	0.493
668	0.493	0.433	0.493
R669C	0.500	0.440	0.500
670	0.523	0.463	0.523
671	0.477	0.477	0.477
In the region 672-714 there are no changes			
715	0.413	0.413	0.413
716	0.350	0.373	0.350
C717Y	0.340	0.363	0.340
718	0.390	0.413	0.390
719	0.427	0.427	0.427
In the region 720-792 there are no changes			
793	0.410	0.410	0.410
794	0.450	0.397	0.450
S795C	0.447	0.393	0.447
796	0.393	0.340	0.393
797	0.400	0.400	0.400
798	0.463	0.463	0.463
799	0.510	0.510	0.510
800	0.463	0.433	0.463
V801M	0.470	0.440	0.470
802	0.403	0.373	0.403
803	0.377	0.377	0.377
In the region 804-826 there are no changes			
827	0.457	0.457	0.457
828	0.453	0.430	0.453
I829V	0.453	0.430	0.403
G830V	0.503	0.480	0.453
831	0.527	0.477	0.477
832	0.470	0.470	0.470

833	0.420	0.420	0.420
834	0.373	0.373	0.373
835	0.403	0.380	0.403
I836V	0.430	0.407	0.430
837	0.463	0.440	0.463
838	0.463	0.463	0.463
In the region 839-846 there are no changes			
847	0.487	0.487	0.487
848	0.460	0.407	0.460
I849M	0.477	0.423	0.477
850	0.427	0.373	0.427
851	0.427	0.427	0.427
852	0.397	0.410	0.397
Y853N	0.473	0.487	0.473
854	0.423	0.437	0.423
855	0.447	0.447	0.447
In the region 856-890 there are no changes			
891	0.470	0.470	0.470
892	0.517	0.467	0.493
S893A/ S893T	0.530	0.480	0.507
894	0.507	0.457	0.483
895	0.490	0.490	0.490
In the region 896-904 there are no changes			
905	0.427	0.427	0.427
906	0.453	0.427	0.453
V907F	0.407	0.380	0.407
908	0.430	0.403	0.430
909	0.423	0.423	0.423
In the region 910-925 there are no changes			

926	0.463	0.463	0.463
927	0.440	0.470	0.440
Y928S	0.487	0.517	0.487
929	0.470	0.500	0.470
930	0.500	0.500	0.500
In the region 931-975 there are no changes			
976	0.377	0.377	0.377
977	0.357	0.373	0.357
F978A	0.403	0.420	0.403
979	0.393	0.410	0.443
A980P	0.420	0.420	0.470
981	0.380	0.430	0.430
982	0.363	0.363	0.363
983	0.413	0.413	0.413
984	0.403	0.403	0.403
985	0.400	0.430	0.400
M986V	0.340	0.370	0.340
987	0.350	0.380	0.350
988	0.430	0.430	0.430
In the region 989-996 there are no changes			
997	0.480	0.480	0.480
998	0.430	0.457	0.430
A999T	0.417	0.443	0.417
1000	0.397	0.423	0.397
1001	0.433	0.433	0.433
In the region 1002-1048 there are no changes			
1049	0.493	0.493	0.493
1050	0.493	0.443	0.493
P1051A	0.453	0.403	0.453
1052	0.423	0.373	0.423

1053	0.417	0.417	0.417
In the region 1054-1104 there are no changes			
1105	0.407	0.407	0.407
1106	0.447	0.453	0.447
Q1107P	0.397	0.403	0.470
W1108R	0.390	0.397	0.463
1109	0.403	0.403	0.477
1110	0.420	0.420	0.420
1111	0.403	0.403	0.403
In the region 1112-1138 there are no changes			
1139	0.437	0.437	0.437
1140	0.430	0.407	0.430
S1141T	0.463	0.440	0.463
1142	0.500	0.477	0.500
1143	0.497	0.497	0.497
In the region 1144-1248 there are no changes			
1249	0.510	0.510	0.510
1250	0.487	0.510	0.487
V1251I	0.463	0.487	0.463
1252	0.453	0.477	0.453
1253	0.430	0.430	0.430
1254	0.453	0.453	0.453
1255	0.433	0.443	0.433
T1256K	0.433	0.443	0.433
1257	0.417	0.427	0.417
1258	0.433	0.433	0.433
In the region 1259-1279 there are no changes			

Table S3. Changes in local hydropathicity resulting from amino acid substitutions. Hydropathicity values were calculated using the ProtScale tool with a five-residue sliding window. The window centered on the substituted residue is highlighted in light orange for the wild-type protein and in blue for the mutant proteins. Regions unaffected by the substitutions are omitted.

Aminoacid number and mutation	Hidropathicity values for 5 aminoacids windows for wild-type protein	Hidropathicity values for allelic sequences with mutations: N15D, N44S, H61Y, A80G, M89T, F103L, Y116C, N183S, I261V, L305P, S400N, R492C, E566K, R580P, R593C, A599T, L662, R669C, C717Y, S795C, V801M, I829V, I836V, I849M, S893A, V907F, Y928S, F978A, M986V, A999T, P1051A, Q1107P, S1141T, V1251I, T1256K	Hidropathicity values for allelic sequence with mutations: F17L, A80E, G185V, G830V, Y853N, S893T, A980P, W1108R	Hidropathicity values for allelic sequences with mutations: N21D, I186N
In the region 3-11 there are no changes				
12	-2.060	-2.060	-2.060	-2.060
13	-2.680	-2.680	-2.680	-2.680
14	-2.480	-2.480	-2.480	-2.480
N15D	-1.140	-1.140	-0.940	-1.140
16	-1.140	-1.140	-0.940	-1.140
F17L	0.400	0.400	0.600	0.400
18	0.400	0.400	0.600	0.400
19	-0.860	-0.860	-0.660	-0.860
20	-2.200	-2.200	-2.200	-2.200
N21D	-1.580	-1.580	-1.580	-1.580
22	-3.040	-3.040	-3.040	-3.040
23	-3.120	-3.120	-3.120	-3.120
24	-3.120	-3.120	-3.120	-3.120
In the region 25-40 there are no changes				
41	-0.380	-0.380	-0.380	-0.380
42	-1.460	-0.920	-1.460	-1.460
43	-2.200	-1.660	-2.200	-2.200
N44S	-0.540	-0.000	-0.540	-0.540
45	-0.980	-0.440	-0.980	-0.980
46	-1.600	-1.060	-1.600	-1.600
47	-0.140	-0.140	-0.140	-0.140

		In the region 48-57 there are no changes		
58	3.280	3.280	3.280	3.280
59	1.880	2.260	1.880	1.880
60	1.440	1.820	1.440	1.440
H61Y	1.440	1.820	1.440	1.440
62	0.460	0.840	0.460	0.460
63	0.320	0.700	0.320	0.320
64	0.640	0.640	0.640	0.640
		In the region 65-76 there are no changes		
77	1.000	1.000	1.000	1.000
78	0.980	0.540	-0.080	0.980
79	0.420	-0.020	-0.640	0.420
A80G/ A80E	1.480	1.040	0.420	1.480
81	0.500	0.060	-0.560	0.500
82	-0.760	-1.200	-1.820	-0.760
83	-0.360	-0.360	-0.360	-0.360
		In the region 84-85 there are no changes		
86	-0.580	-0.580	-0.580	-0.580
87	0.500	-0.020	0.500	0.500
88	-0.420	-0.940	-0.420	-0.420
M89T	-0.420	-0.940	-0.420	-0.420
90	1.180	0.660	1.180	1.180
91	0.280	-0.240	0.280	0.280
92	-0.800	-0.800	-0.800	-0.800
		In the region 93-99 there are no changes		
100	-0.720	-0.720	-0.720	-0.720
101	-1.060	-0.860	-1.060	-1.060
102	0.200	0.400	0.200	0.200
F103L	1.280	1.480	1.280	1.280

104	0.720	0.920	0.720	0.720
105	1.560	1.760	1.560	1.560
106	0.300	0.300	0.300	0.300
In the region 107-112 there are no changes				
113	-0.560	-0.560	-0.560	-0.560
114	-1.200	-0.440	-1.200	-1.200
115	-1.320	-0.560	-1.320	-1.320
Y116C	-0.680	0.080	-0.680	-0.680
117	-0.580	0.180	-0.580	-0.580
118	-1.020	-0.260	-1.020	-1.020
119	0.140	0.140	0.140	0.140
In the region 120-179 there are no changes				
180	0.100	0.100	0.100	0.100
181	0.100	0.640	0.100	0.100
182	-1.440	-0.900	-1.440	-1.440
N183S	-1.360	-0.820	-0.440	-1.360
184	0.320	0.860	1.240	-2.260
G185V	-0.660	-0.120	0.260	-2.340
I186N	-0.660	-0.660	0.260	-1.360
187	-0.740	-0.740	0.180	-0.740
188	0.240	0.240	0.240	-0.280
189	-0.740	-0.740	-0.740	-0.740
In the region 190-257 there are no changes				
258	1.620	1.620	1.620	1.620
259	3.220	3.160	3.220	3.220
260	1.480	1.420	1.480	1.480
I261V	0.580	0.520	0.580	0.580
262	1.060	1.000	1.060	1.060
263	1.600	1.540	1.600	1.600
264	1.060	1.060	1.060	1.060

In the region 265-301 there are no changes				
302	1.960	1.960	1.960	1.960
303	2.800	1.720	2.800	2.800
304	3.340	2.260	3.340	3.340
L305P	2.720	1.640	2.720	2.720
306	2.520	1.440	2.520	2.520
307	1.600	0.520	1.600	1.600
308	0.580	0.580	0.580	0.580
In the region 308-396 there are no changes				
397	-0.840	-0.840	-0.840	-0.840
398	-0.100	-0.640	-0.100	-0.100
399	0.340	-0.200	0.340	0.340
S400N	-0.820	-1.360	-0.820	-0.820
401	-0.340	-0.880	-0.340	-0.340
402	-1.800	-2.340	-1.800	-1.800
403	-2.420	-2.420	-2.420	-2.420
In the region 404-488 there are no changes				
489	-1.040	-1.040	-1.040	-1.040
490	-1.240	0.160	-1.240	-1.240
491	-2.840	-1.440	-2.840	-2.840
R492C	-2.640	-1.240	-2.640	-2.640
493	-1.540	-0.140	-1.540	-1.540
494	-1.600	-0.200	-1.600	-1.600
495	-0.320	-0.320	-0.320	-0.320
In the region 496-562 there are no changes				
563	-0.940	-0.940	-0.940	-0.940
564	-2.400	-2.480	-2.400	-2.400
565	-1.340	-1.420	-1.340	-1.340
E566K	-0.360	-0.440	-0.360	-0.360
567	1.180	1.100	1.180	1.180

568	0.640	0.560	0.640	0.640
569	2.180	2.180	2.180	2.180
In the region 570-576 there are no changes				
577	-2.180	-2.180	-2.180	-2.180
578	-2.300	-1.720	-2.300	-2.300
579	-2.800	-2.220	-2.800	-2.800
R580P	-2.040	-1.460	-2.040	-2.040
581	-0.360	0.220	-0.360	-0.360
582	0.560	1.140	0.560	0.560
583	2.360	2.360	2.360	2.360
In the region 584-589 there are no changes				
590	0.400	0.400	0.400	0.400
591	0.400	1.800	0.400	0.400
592	-1.060	0.340	-1.060	-1.060
R593C	-0.540	0.860	-0.540	-0.540
594	-1.100	0.300	-1.100	-1.100
595	-1.100	0.300	-1.100	-1.100
596	0.700	0.700	0.700	0.700
597	1.760	1.260	1.760	1.760
598	1.320	0.820	1.320	1.320
A599T	2.580	2.080	2.580	2.580
600	1.040	0.540	1.040	1.040
601	-0.560	-1.060	-0.560	-0.560
602	-1.000	-1.000	-1.000	-1.000
In the region 603-658 there are no changes				
659	-2.080	-2.080	-2.080	-2.080
660	-0.620	-2.280	-0.620	-0.620
661	0.440	-1.220	0.440	0.440
L662R	0.440	-1.220	0.440	0.440
663	-0.180	-1.840	-0.180	-0.180

664	-0.920	-2.580	-0.920	-0.920
665	-1.840	-1.840	-1.840	-1.840
666	-2.880	-2.880	-2.880	-2.880
667	-2.880	-1.480	-2.880	-2.880
668	-3.000	-1.600	-3.000	-3.000
R669C	-2.260	-0.860	-2.260	-2.260
670	-1.260	0.140	-1.260	-1.260
671	-2.020	-0.620	-2.020	-2.020
672	-1.200	-1.200	-1.200	-1.200
In the region 673-713 there are no changes				
714	3.000	3.000	3.000	3.000
715	2.660	1.900	2.660	2.660
716	2.180	1.420	2.180	2.180
C717Y	3.160	2.400	3.160	3.160
718	3.220	2.460	3.220	3.220
719	1.960	1.200	1.960	1.960
720	1.380	1.380	1.380	1.380
In the region 721-791 there are no changes				
792	0.620	0.620	0.620	0.620
793	0.720	1.380	0.720	0.720
794	0.720	1.380	0.720	0.720
S795C	0.640	1.300	0.640	0.640
796	-0.820	-0.160	-0.820	-0.820
797	-0.620	0.040	-0.620	-0.620
798	-1.160	-1.160	-1.160	-1.160
799	-0.700	-1.160	-0.700	-0.700
800	-1.620	-2.080	-1.620	-1.620
V801M	-0.900	-1.360	-0.900	-0.900
802	0.360	-0.100	0.360	0.360

803	0.360	-0.100	0.360	0.360
804	-1.180	-1.180	-1.180	-1.180
In the region 805-825 there are no changes				
826	-0.360	-0.360	-0.360	-0.360
827	1.240	1.180	1.240	1.240
828	0.320	0.260	1.240	0.320
I829V	0.940	0.880	1.860	0.940
G830V	0.120	0.060	1.040	0.120
831	0.520	0.460	1.440	0.520
832	-0.020	0.900	0.900	-0.020
833	0.900	0.900	0.900	0.900
834	1.960	1.900	1.960	1.960
835	2.720	2.660	2.720	2.720
I836V	1.260	1.200	1.260	1.260
837	0.200	0.140	0.200	0.200
838	0.260	0.200	0.260	0.260
839	-0.280	-0.280	-0.280	-0.280
In the region 838-845 there are no changes				
846	1.500	1.500	1.500	1.500
847	2.480	1.960	2.480	2.480
848	2.460	1.940	2.460	2.460
I849M	3.100	2.580	3.100	3.100
850	3.100	2.580	3.100	3.100
851	1.940	1.420	1.500	1.940
852	0.960	0.960	0.520	0.960
Y853N	0.940	0.940	0.500	0.940
854	-0.320	-0.320	-0.760	-0.320
855	-0.460	-0.460	-0.900	-0.460
856	-0.340	-0.340	-0.340	-0.340
In the region 857-889 there are no changes				

890	-1.500	-1.500	-1.500	-1.500
891	-0.880	-0.360	-0.860	-0.880
892	-0.260	0.260	-0.240	-0.260
S893A/ S893T	-1.800	-1.280	-1.780	-1.800
894	-0.200	0.320	-0.180	-0.200
895	0.240	0.760	0.260	0.240
896	0.260	0.260	0.260	0.260
In the region 897-903 there are no changes				
904	-1.880	-1.880	-1.880	-1.880
905	-0.340	-0.620	-0.340	-0.340
906	1.200	0.920	1.200	1.200
V907F	0.480	0.200	0.480	0.480
908	2.140	1.860	2.140	2.140
909	2.140	1.860	2.140	2.140
910	0.600	0.600	0.600	0.600
In the region 909-924 there are no changes				
925	0.420	0.420	0.420	0.420
926	0.320	0.420	0.320	0.320
927	-1.340	-1.240	-1.340	-1.340
Y928S	-1.340	-1.240	-1.340	-1.340
929	-2.340	-2.240	-2.340	-2.340
930	-1.260	-1.160	-1.260	-1.260
931	-1.900	-1.900	-1.900	-1.900
In the region 930-974 there are no changes				
975	2.500	2.500	2.500	2.500
976	3.760	3.560	3.760	3.560
977	2.760	2.560	2.760	2.560
F978A	2.360	2.160	1.680	2.160
979	2.440	2.240	1.760	2.240

A980P	2.440	2.240	1.760	2.240
981	2.440	2.440	1.760	2.440
982	2.520	2.520	1.840	2.520
983	2.520	2.520	2.520	2.520
984	2.060	2.520	2.060	2.060
985	1.580	2.040	1.580	1.580
M986V	1.860	2.320	1.860	1.860
987	1.860	2.320	1.860	1.860
988	0.800	1.260	0.800	0.800
989	1.260	1.260	1.260	1.260
In the region 990-995 there are no changes				
996	-0.360	-0.360	-0.360	-0.360
997	-0.560	-1.060	-0.560	-0.560
998	-1.700	-2.200	-1.700	-1.700
A999T	-1.020	-1.520	-1.020	-1.020
1000	-1.100	-1.600	-1.100	-1.100
1001	0.060	-0.440	0.060	0.060
1002	-0.460	-0.460	-0.460	-0.460
In the region 1003-1047 there are no changes				
1048	-1.160	-1.160	-1.160	-1.160
1049	-1.340	-0.660	-1.340	-1.340
1050	0.400	1.080	0.400	0.400
P1051A	1.480	2.160	1.480	1.480
1052	1.480	2.160	1.480	1.480
1053	0.500	1.180	0.500	0.500
1054	1.580	1.580	1.580	1.580
In the region 1055-1103 there are no changes				
1104	-0.780	-0.780	-0.780	-0.780
1105	-0.700	-0.320	-0.700	-0.700
1106	0.020	0.400	-0.700	0.020

Q1107P	0.020	0.400	-0.700	0.020
W1108R	-0.180	0.200	-0.900	-0.180
1109	-0.660	-0.280	-1.380	-0.660
1110	-0.600	-0.600	-1.320	-0.600
1111	0.340	0.340	0.340	0.340
	In the region 1112-1137 there are no changes			
1138	-0.080	-0.080	-0.080	-0.080
1139	0.460	0.480	0.460	0.460
1140	-0.080	-0.060	-0.080	-0.080
S1141T	0.120	0.140	0.120	0.120
1142	-1.420	-1.400	-1.420	-1.420
1143	-1.360	-1.340	-1.360	-1.360
1144	-0.360	-0.360	-0.360	-0.360
	In the region 1145-1247 there are no changes			
1248	-1.820	-1.820	-1.820	-1.820
1249	-1.540	-1.480	-1.540	-1.540
1250	-1.620	-1.560	-1.620	-1.620
V1251I	-1.620	-1.560	-1.620	-1.620
1252	-2.180	-2.120	-2.180	-2.180
1253	-1.360	-1.300	-1.360	-1.360
1254	-2.340	-2.980	-2.340	-2.340
1255	-2.200	-2.840	-2.200	-2.200
T1256K	-2.200	-2.840	-2.200	-2.200
1257	-2.260	-2.900	-2.260	-2.260
1258	-1.420	-2.060	-1.420	-1.420
1259	-0.520	-0.520	-0.520	-0.520
	In the region 1260-1278 there are no changes			

Table S4. Changes in local flexibility resulting from amino acid substitutions. Flexibility values were calculated using the ProtScale tool with a five-residue sliding window. The window centered on the substituted residue is highlighted in light orange for the wild-type protein and in blue for the mutant proteins. Regions unaffected by the substitutions are omitted.

Aminoacid number and mutation	Flexibility values for 5 aminoacids windows for wild-type protein	Flexibility values for allelic sequence with N15D, N44S, H61Y, A80E, M89T, F103L, Y116C, N183S, I261V, L305P, S400N, R492C, E566K, R580P, R593C, A599T, L662R, R669C, C717Y, S795C, V801M, I829V, I836V, I849M, S893A, V907F, Y928S, F978A, M986V, A999T, P1051A, Q1107P, S1141T, V1251I and T1256K	Flexibility values for allelic sequence with F17L, A80G, G185V, G830V, Y853N, S893T, A980P and W1108R	Flexibility values for allelic sequence with N21D and I186N
In the region 3-11 there are no changes				
12	0.462	0.462	0.462	0.462
13	0.446	0.456	0.446	0.446
14	0.436	0.446	0.436	0.436
N15D	0.404	0.414	0.416	0.404
16	0.404	0.414	0.416	0.404
F17L	0.384	0.394	0.396	0.384
18	0.384	0.384	0.396	0.384
19	0.414	0.414	0.426	0.424
20	0.446	0.446	0.446	0.456
N21D	0.454	0.454	0.454	0.464
22	0.480	0.480	0.480	0.490
23	0.482	0.482	0.482	0.492
24	0.492	0.492	0.492	0.492
In the region 25-40 there are no changes				
41	0.414	0.414	0.414	0.414
42	0.446	0.456	0.446	0.446
43	0.446	0.456	0.446	0.446
N44S	0.414	0.424	0.414	0.414
45	0.432	0.442	0.432	0.432
46	0.424	0.434	0.424	0.424

47	0.406	0.406	0.406	0.406
In the region 48-57 there are no changes				
58	0.402	0.402	0.402	0.402
59	0.392	0.412	0.392	0.392
60	0.428	0.448	0.428	0.428
H61Y	0.428	0.448	0.428	0.428
62	0.444	0.464	0.444	0.444
63	0.426	0.446	0.426	0.426
64	0.464	0.464	0.464	0.464
In the region 65-76 there are no changes				
77	0.404	0.404	0.404	0.404
78	0.416	0.444	0.452	0.416
79	0.420	0.448	0.456	0.420
A80E/G	0.390	0.418	0.426	0.390
81	0.406	0.434	0.442	0.406
82	0.436	0.464	0.472	0.436
83	0.438	0.438	0.438	0.438
In the region 84-85 there are no changes				
86	0.442	0.442	0.442	0.442
87	0.410	0.438	0.410	0.410
88	0.438	0.466	0.438	0.438
M89T	0.430	0.458	0.430	0.430
90	0.420	0.448	0.420	0.420
91	0.434	0.462	0.434	0.434
92	0.466	0.466	0.466	0.466
In the region 93-99 there are no changes				
100	0.482	0.482	0.482	0.482
101	0.452	0.464	0.452	0.452
102	0.422	0.434	0.422	0.422

F103L	0.380	0.392	0.380	0.380
104	0.384	0.396	0.384	0.384
105	0.350	0.362	0.350	0.350
106	0.388	0.388	0.388	0.388
In the region 107-112 there are no changes				
113	0.410	0.410	0.410	0.410
114	0.434	0.420	0.434	0.434
115	0.430	0.416	0.430	0.430
Y116C	0.408	0.394	0.408	0.408
117	0.426	0.412	0.426	0.426
118	0.462	0.448	0.462	0.462
119	0.470	0.470	0.470	0.470
In the region 120-179 there are no changes				
180	0.468	0.468	0.468	0.468
181	0.458	0.468	0.458	0.458
182	0.480	0.490	0.480	0.480
N183S	0.486	0.496	0.456	0.486
184	0.484	0.494	0.454	0.484
G185V	0.500	0.510	0.470	0.500
I186N	0.510	0.510	0.480	0.510
187	0.504	0.504	0.474	0.504
188	0.488	0.488	0.488	0.488
189	0.504	0.504	0.504	0.504
In the region 190-257 there are no changes				
258	0.396	0.396	0.396	0.396
259	0.388	0.374	0.388	0.388
260	0.416	0.402	0.416	0.416
I261V	0.430	0.416	0.430	0.430
262	0.436	0.422	0.436	0.436

263	0.456	0.442	0.456	0.456
264	0.436	0.436	0.436	0.436
In the region 265-301 there are no changes				
302	0.388	0.388	0.388	0.388
303	0.354	0.382	0.354	0.354
304	0.374	0.402	0.374	0.374
L305P	0.386	0.414	0.386	0.386
306	0.396	0.424	0.396	0.396
307	0.424	0.452	0.424	0.424
308	0.434	0.434	0.434	0.434
In the region 309-396 there are no changes				
397	0.402	0.402	0.402	0.402
398	0.398	0.388	0.398	0.398
399	0.390	0.380	0.390	0.390
S400N	0.414	0.404	0.414	0.414
401	0.452	0.442	0.452	0.452
402	0.496	0.486	0.496	0.496
403	0.488	0.488	0.488	0.488
In the region 404-488 there are no changes				
489	0.482	0.482	0.482	0.482
490	0.496	0.460	0.496	0.496
491	0.504	0.468	0.504	0.504
R492C	0.490	0.454	0.490	0.490
493	0.484	0.448	0.484	0.484
494	0.464	0.428	0.464	0.464
495	0.418	0.418	0.418	0.418
In the region 496-562 there are no changes				
563	0.466	0.466	0.466	0.466
564	0.492	0.486	0.492	0.492

565	0.462	0.456	0.462	0.462
E566K	0.452	0.446	0.452	0.452
567	0.430	0.424	0.430	0.430
568	0.426	0.420	0.426	0.426
569	0.404	0.404	0.404	0.404
		In the region 570-576 there are no changes		
577	0.474	0.474	0.474	0.474
578	0.486	0.482	0.486	0.486
579	0.502	0.498	0.502	0.502
R580P	0.484	0.480	0.484	0.484
581	0.482	0.478	0.482	0.482
582	0.452	0.448	0.452	0.452
583	0.438	0.438	0.438	0.438
		In the region 584-589 there are no changes		
590	0.448	0.448	0.448	0.448
591	0.448	0.412	0.448	0.448
592	0.466	0.430	0.466	0.466
R593C	0.436	0.400	0.436	0.436
594	0.450	0.414	0.450	0.450
595	0.450	0.414	0.450	0.450
596	0.436	0.436	0.436	0.436
597	0.416	0.432	0.416	0.416
598	0.452	0.468	0.452	0.452
A599T	0.412	0.428	0.412	0.412
600	0.436	0.452	0.436	0.436
601	0.446	0.462	0.446	0.446
602	0.482	0.482	0.482	0.482
		In the region 603-658 there are no changes		
659	0.514	0.514	0.514	0.514

660	0.486	0.518	0.486	0.486
661	0.476	0.508	0.476	0.476
L662R	0.476	0.508	0.476	0.476
663	0.468	0.500	0.468	0.468
664	0.472	0.504	0.472	0.472
665	0.500	0.500	0.500	0.500
666	0.496	0.496	0.496	0.496
667	0.496	0.460	0.496	0.496
668	0.508	0.472	0.508	0.508
R669C	0.504	0.468	0.504	0.504
670	0.480	0.444	0.480	0.480
671	0.498	0.462	0.498	0.498
672	0.500	0.500	0.500	0.500
In the region 673-713 there are no changes				
714	0.404	0.404	0.404	0.404
715	0.396	0.410	0.396	0.396
716	0.390	0.404	0.390	0.390
C717Y	0.374	0.388	0.374	0.374
718	0.388	0.402	0.388	0.388
719	0.418	0.432	0.418	0.418
720	0.456	0.456	0.456	0.456
In the region 719-791 there are no changes				
792	0.390	0.390	0.390	0.390
793	0.408	0.376	0.408	0.408
794	0.408	0.376	0.408	0.408
S795C	0.404	0.372	0.404	0.404
796	0.448	0.416	0.448	0.448
797	0.440	0.408	0.440	0.440
798	0.440	0.440	0.440	0.440

799	0.458	0.440	0.458	0.458
800	0.486	0.468	0.486	0.486
V801M	0.442	0.424	0.442	0.442
802	0.406	0.388	0.406	0.406
803	0.406	0.388	0.406	0.406
804	0.430	0.430	0.430	0.430
In the region 805-825 there are no changes				
826	0.450	0.450	0.450	0.450
827	0.444	0.430	0.444	0.444
828	0.474	0.460	0.444	0.474
I829V	0.482	0.468	0.452	0.482
G830V	0.480	0.466	0.450	0.480
831	0.482	0.468	0.452	0.482
832	0.462	0.462	0.432	0.462
833	0.432	0.432	0.432	0.432
834	0.422	0.408	0.422	0.422
835	0.404	0.390	0.404	0.404
I836V	0.428	0.414	0.428	0.428
837	0.448	0.434	0.448	0.448
838	0.462	0.448	0.462	0.462
839	0.442	0.442	0.442	0.442
In the region 840-845 there are no changes				
846	0.488	0.488	0.488	0.488
847	0.472	0.440	0.472	0.472
848	0.486	0.454	0.486	0.486
I849M	0.440	0.408	0.440	0.440
850	0.440	0.408	0.440	0.440
851	0.432	0.400	0.440	0.432
852	0.448	0.448	0.456	0.448

Y853N	0.408	0.408	0.416	0.408
854	0.444	0.444	0.452	0.444
855	0.426	0.426	0.434	0.426
856	0.430	0.430	0.430	0.430
In the region 857-889 there are no changes				
890	0.476	0.476	0.476	0.476
891	0.484	0.454	0.470	0.484
892	0.492	0.462	0.478	0.492
S893A/T	0.512	0.482	0.498	0.512
894	0.504	0.474	0.490	0.504
895	0.468	0.438	0.454	0.468
896	0.454	0.454	0.454	0.454
In the region 897-903 there are no changes				
904	0.448	0.448	0.448	0.448
905	0.426	0.410	0.426	0.426
906	0.412	0.396	0.412	0.412
V907F	0.452	0.436	0.452	0.452
908	0.420	0.404	0.420	0.420
909	0.420	0.404	0.420	0.420
910	0.440	0.440	0.440	0.440
In the region 911-924 there are no changes				
925	0.454	0.454	0.454	0.454
926	0.436	0.454	0.436	0.436
927	0.468	0.486	0.468	0.468
Y928S	0.462	0.480	0.462	0.462
929	0.486	0.504	0.486	0.486
930	0.458	0.476	0.458	0.458
931	0.480	0.480	0.480	0.480
In the region 932-974 there are no changes				

975	0.406	0.406	0.406	0.406
976	0.366	0.376	0.366	0.366
977	0.390	0.400	0.390	0.390
F978A	0.388	0.398	0.418	0.388
979	0.392	0.402	0.422	0.392
A980P	0.392	0.402	0.422	0.392
981	0.392	0.392	0.422	0.392
982	0.398	0.398	0.428	0.398
983	0.398	0.398	0.398	0.398
984	0.380	0.398	0.380	0.380
985	0.374	0.392	0.374	0.374
M986V	0.390	0.408	0.390	0.390
987	0.390	0.408	0.390	0.390
988	0.416	0.434	0.416	0.416
989	0.434	0.434	0.434	0.434
In the region 990-995 there are no changes				
996	0.422	0.422	0.422	0.422
997	0.432	0.448	0.432	0.432
998	0.454	0.470	0.454	0.454
A999T	0.424	0.440	0.424	0.424
1000	0.416	0.432	0.416	0.416
1001	0.424	0.440	0.424	0.424
1002	0.454	0.454	0.454	0.454
In the region 1003-1047 there are no changes				
1048	0.490	0.490	0.490	0.490
1049	0.504	0.474	0.504	0.504
1050	0.476	0.446	0.476	0.476
P1051A	0.448	0.418	0.448	0.448
1052	0.444	0.414	0.444	0.444

1053	0.460	0.430	0.460	0.460
1054	0.432	0.432	0.432	0.432
In the region 1055-1103 there are no changes				
1104	0.444	0.444	0.444	0.444
1105	0.448	0.452	0.448	0.448
1106	0.404	0.408	0.448	0.404
Q1107P	0.404	0.408	0.462	0.404
W1108R	0.418	0.422	0.456	0.418
1109	0.412	0.416	0.422	0.412
1110	0.378	0.378	0.390	0.378
1111	0.390	0.390	0.390	0.390
In the region 1112-1137 there are no changes				
1138	0.456	0.456	0.456	0.456
1139	0.466	0.452	0.466	0.466
1140	0.462	0.448	0.462	0.462
S1141T	0.456	0.442	0.456	0.456
1142	0.478	0.464	0.478	0.478
1143	0.492	0.478	0.492	0.492
1144	0.468	0.468	0.468	0.468
In the region 1145-1247 there are no changes				
1248	0.466	0.466	0.466	0.466
1249	0.482	0.496	0.482	0.482
1250	0.478	0.492	0.478	0.478
V1251I	0.486	0.500	0.486	0.486
1252	0.442	0.456	0.442	0.442
1253	0.444	0.458	0.444	0.444
1254	0.454	0.460	0.454	0.454
1255	0.424	0.430	0.424	0.424
T1256K	0.422	0.428	0.422	0.422

1257	0.456	0.462	0.456	0.456
1258	0.422	0.428	0.422	0.422
1259	0.408	0.408	0.408	0.408

In the region 1260-1278 there are no changes

Table S5. Changes in local hydropathicity resulting from amino acid substitutions. Hydropathicity values were calculated using the ProtScale tool with a nine-residue sliding window. The window centered on the substituted residue is highlighted in light orange for the wild-type protein and in blue for the mutant proteins. Regions unaffected by the substitutions are omitted.

Aminoacid number and mutation	Hidropathicity values for 9 aminoacids windows for wild-type protein	Hidropathicity values for allelic sequence with N15D, N44S, H61Y, A80E, M89T, F103L, Y116C, N183S, I261V, L305P, S400N, R492C, E566K, R580P, R593C, L662R, C717Y, S795C, I829V, I849M, S893A, V907F, Y928S, F978A, A999T, P1051A, Q1107P, S1141T and V1251I	Hidropathicity values for allelic sequence with F17L, A80G, G185V, A599T, R669C, V801M, G830V, Y853N, S893T, A980P, W1108R and T1256K	Hidropathicity values for allelic sequence with N21D, I186N, I836V and M986V
In the region 5-9 there are no changes				
10	-2.467	-2.467	-2.467	-2.467
11	-2.467	-2.467	-2.467	-2.467
12	-1.656	-1.656	-1.656	-1.656
13	-0.956	-0.956	-0.844	-0.956
14	-1.344	-1.344	-1.233	-1.344
N15D	-0.878	-0.878	-0.767	-0.878
16	-1.467	-1.467	-1.356	-1.467
F17L	-1.422	-1.422	-1.311	-1.422
18	-1.422	-1.422	-1.311	-1.422
19	-1.078	-1.078	-0.967	-1.078
20	-1.078	-1.078	-0.967	-1.078
N21D	-1.822	-1.822	-1.711	-1.822
22	-2.522	-2.522	-2.522	-2.522
23	-2.522	-2.522	-2.522	-2.522
24	-3.378	-3.378	-3.378	-3.378
25	-3.378	-3.378	-3.378	-3.378
26	-3.422	-3.422	-3.422	-3.422
In the region 27-38 there are no changes				
39	0.389	0.389	0.389	0.389
40	0.089	0.389	0.089	0.089

41	-0.478	-0.178	-0.478	-0.478
42	-0.367	-0.067	-0.367	-0.367
43	-0.667	-0.367	-0.667	-0.667
N44S	-1.311	-1.011	-1.311	-1.311
45	-1.200	-0.900	-1.200	-1.200
46	-0.844	-0.544	-0.844	-0.844
47	-0.489	-0.189	-0.489	-0.489
48	0.067	0.367	0.067	0.067
49	0.922	0.922	0.922	0.922
In the region 48-55 there are no changes				
56	2.633	2.633	2.633	2.633
57	1.811	2.022	1.811	1.811
58	1.300	1.511	1.300	1.300
59	1.544	1.756	1.544	1.544
60	1.578	1.789	1.578	1.578
H61Y	1.578	1.789	1.578	1.578
62	1.200	1.411	1.200	1.200
63	1.422	1.633	1.422	1.422
64	1.133	1.344	1.133	1.133
65	0.844	1.056	0.844	0.844
66	1.622	1.622	1.622	1.622
In the region 67-74 there are no changes				
75	0.900	0.900	0.900	0.900
76	0.633	0.044	0.389	0.633
77	-0.067	-0.656	-0.311	-0.067
78	0.178	-0.411	-0.067	0.178
79	0.522	-0.067	0.278	0.522
A80E/G	-0.078	-0.667	-0.322	-0.078
81	0.422	-0.167	0.178	0.422

82	0.422	-0.167	0.178	0.422
83	-0.467	-1.056	-0.711	-0.467
84	-0.356	-0.944	-0.600	-0.356
85	-0.344	-0.633	-0.344	-0.344
86	-0.044	-0.333	-0.044	-0.044
87	-0.633	-0.922	-0.633	-0.633
88	-0.089	-0.378	-0.089	-0.089
M89T	0.222	-0.067	0.222	0.222
90	-0.589	-0.878	-0.589	-0.589
91	-0.700	-0.989	-0.700	-0.700
92	-0.400	-0.689	-0.400	-0.400
93	-1.211	-1.500	-1.211	-1.211
94	-0.922	-0.922	-0.922	-0.922
In the region 95-97 there are no changes				
98	-1.767	-1.767	-1.767	-1.767
99	-1.067	-0.956	-1.067	-1.067
100	-0.256	-0.144	-0.256	-0.256
101	0.044	0.156	0.044	0.044
102	0.044	0.156	0.044	0.044
F103L	-0.033	0.078	-0.033	-0.033
104	-0.033	0.078	-0.033	-0.033
105	-0.033	0.078	-0.033	-0.033
106	-0.344	-0.233	-0.344	-0.344
107	-0.089	0.022	-0.089	-0.089
108	-0.478	-0.478	-0.478	-0.478
In the region 109-110 there are no changes				
111	-1.056	-1.056	-1.056	-1.056
112	-1.622	-1.200	-1.622	-1.622
113	-1.378	-0.956	-1.378	-1.378

114	-1.133	-0.711	-1.133	-1.133
115	-0.833	-0.411	-0.833	-0.833
Y116C	-1.089	-0.667	-1.089	-1.089
117	-0.511	-0.089	-0.511	-0.511
118	-0.056	0.367	-0.056	-0.056
119	0.289	0.711	0.289	0.289
120	0.044	0.467	0.044	0.044
121	0.656	0.656	0.656	0.656
In the region 122-177 there are no changes				
178	-0.489	-0.489	-0.489	-0.489
179	-0.378	-0.078	-0.378	-0.378
180	-1.189	-0.889	-1.189	-1.189
181	-1.156	-0.856	-0.644	-1.156
182	-0.267	0.033	0.244	-1.156
N183S	0.078	0.378	0.589	-0.811
184	-0.778	-0.478	-0.267	-1.667
G185V	-1.122	-0.822	-0.611	-2.011
I186N	-0.189	0.111	0.322	-1.078
187	-0.733	-0.433	-0.222	-1.622
188	-0.133	-0.133	0.378	-1.022
189	0.567	0.567	1.078	-0.322
190	0.922	0.922	0.922	0.033
191	0.033	0.033	0.033	0.033
In the region 192-254 there are no changes				
256	1.378	1.378	1.378	1.378
257	1.678	1.644	1.678	1.678
258	0.711	0.678	0.711	0.711
259	0.433	0.400	0.433	0.433
260	1.289	1.256	1.289	1.289

I261V	2.178	2.144	2.178	2.178
262	1.911	1.878	1.911	1.911
263	1.800	1.767	1.800	1.800
264	1.556	1.522	1.556	1.556
265	1.311	1.278	1.311	1.311
266	0.422	0.422	0.422	0.422
In the region 267-299 there are no changes				
300	1.611	1.611	1.611	1.611
301	2.422	1.822	2.422	2.422
302	2.422	1.822	2.422	2.422
303	2.367	1.767	2.367	2.367
304	2.067	1.467	2.067	2.067
L305P	2.022	1.422	2.022	2.022
306	1.678	1.078	1.678	1.678
307	1.678	1.078	1.678	1.678
308	1.789	1.189	1.789	1.789
309	1.567	0.967	1.567	1.567
310	1.456	1.456	1.456	1.456
In the region 311-394 there are no changes				
395	-0.511	-0.511	-0.511	-0.511
396	-0.211	-0.511	-0.211	-0.211
397	-0.778	-1.078	-0.778	-0.778
398	-0.567	-0.867	-0.567	-0.567
399	-0.967	-1.267	-0.967	-0.967
S400N	-0.967	-1.267	-0.967	-0.967
401	-1.011	-1.311	-1.011	-1.011
402	-1.867	-2.167	-1.867	-1.867
403	-1.044	-1.344	-1.044	-1.044
404	-1.789	-2.089	-1.789	-1.789

405	-1.200	-1.200	-1.200	-1.200
In the region 406-486 there are no changes				
487	-0.344	-0.344	-0.344	-0.344
488	-0.767	0.011	-0.767	-0.767
489	-1.656	-0.878	-1.656	-1.656
490	-2.244	-1.467	-2.244	-2.244
491	-1.389	-0.611	-1.389	-1.389
R492C	-1.078	-0.300	-1.078	-1.078
493	-1.367	-0.589	-1.367	-1.367
494	-1.256	-0.478	-1.256	-1.256
495	-1.500	-0.722	-1.500	-1.500
496	-0.956	-0.956	-0.956	-0.956
497	-0.844	-0.844	-0.844	-0.844
In the region 498-560 there are no changes				
561	-0.289	-0.289	-0.289	-0.289
562	-0.878	-0.922	-0.878	-0.878
563	-0.600	-0.644	-0.600	-0.600
564	-0.044	-0.089	-0.044	-0.044
565	0.222	0.178	0.222	0.222
E566K	-0.589	-0.633	-0.589	-0.589
567	0.267	0.222	0.267	0.267
568	0.544	0.500	0.544	0.544
569	1.356	1.311	1.356	1.356
570	1.056	1.011	1.056	1.056
571	1.011	1.011	1.011	1.011
In the region 572-574 there are no changes				
575	-0.511	-0.511	-0.511	-0.511
576	-1.478	-1.156	-1.478	-1.478
577	-1.756	-1.433	-1.756	-1.756

578	-2.256	-1.933	-2.256	-2.256
579	-1.367	-1.044	-1.367	-1.367
R580P	-0.467	-0.144	-0.467	-0.467
581	-0.167	0.156	-0.167	-0.167
582	0.533	0.856	0.533	0.533
583	0.611	0.933	0.611	0.611
584	0.156	0.478	0.156	0.156
585	1.078	1.078	1.078	1.078
In the region 586-587 there are no changes				
588	1.033	1.033	1.033	1.033
589	0.067	0.844	0.067	0.067
590	-0.822	-0.044	-0.822	-0.822
591	-0.822	-0.044	-0.822	-0.822
592	-0.856	-0.078	-0.856	-0.856
R593C	0.111	0.889	0.111	0.111
594	0.189	0.967	0.189	0.189
595	0.478	1.256	0.200	0.478
596	0.511	1.289	0.233	0.511
597	0.356	1.133	0.078	0.356
598	0.467	0.467	0.189	0.467
A599T	0.467	0.467	0.189	0.467
600	0.222	0.222	-0.056	0.222
601	1.078	1.078	0.800	1.078
602	1.111	1.111	0.833	1.111
603	1.078	1.078	0.800	1.078
604	0.489	0.489	0.489	0.489
In the region 605-656 there are no changes				
657	-1.511	-1.511	-1.511	-1.511
658	-1.300	-2.222	-1.300	-1.300

659	-0.711	-1.633	-0.711	-0.711
660	-1.122	-2.044	-1.122	-1.122
661	-1.167	-2.089	-1.167	-1.167
L662R	-1.278	-2.200	-1.278	-1.278
663	-1.278	-2.200	-1.278	-1.278
664	-0.856	-1.778	-0.856	-0.856
665	-1.267	-2.189	-0.489	-1.267
666	-1.678	-2.600	-0.900	-1.678
667	-2.189	-2.189	-1.411	-2.189
668	-2.222	-2.222	-1.444	-2.222
R669C	-2.222	-2.222	-1.444	-2.222
670	-1.833	-1.833	-1.056	-1.833
671	-1.422	-1.422	-0.644	-1.422
672	-1.722	-1.722	-0.944	-1.722
673	-1.444	-1.444	-0.667	-1.444
674	-1.333	-1.333	-1.333	-1.333
In the region 675-711 there are no changes				
712	1.556	1.556	1.556	1.556
713	1.933	1.511	1.933	1.933
714	2.311	1.889	2.311	2.311
715	2.956	2.533	2.956	2.956
716	3.144	2.722	3.144	3.144
C717Y	2.289	1.867	2.289	2.289
718	1.778	1.356	1.778	1.778
719	1.778	1.356	1.778	1.778
720	1.733	1.311	1.733	1.733
721	1.033	0.611	1.033	1.033
722	0.578	0.578	0.578	0.578
In the region 723-789 there are no changes				

790	-0.667	-0.667	-0.667	-0.667
791	-0.322	0.044	-0.322	-0.322
792	0.389	0.756	0.389	0.389
793	0.389	0.756	0.389	0.389
794	0.389	0.756	0.389	0.389
S795C	0.144	0.511	0.144	0.144
796	-0.456	-0.089	-0.456	-0.456
797	-0.456	-0.089	-0.711	-0.456
798	-0.856	-0.489	-1.111	-0.856
799	-0.456	-0.089	-0.711	-0.456
800	-0.056	-0.056	-0.311	-0.056
V801M	-0.656	-0.656	-0.911	-0.656
802	-1.467	-1.467	-1.722	-1.467
803	-1.144	-1.144	-1.400	-1.144
804	-1.189	-1.189	-1.444	-1.189
805	-1.189	-1.189	-1.444	-1.189
806	-1.733	-1.733	-1.733	-1.733
In the region 805-824 there are no changes				
825	0.311	0.311	0.311	0.278
826	0.656	0.278	0.656	0.656
827	0.367	0.622	1.167	0.367
828	-0.333	0.333	0.878	-0.333
I829V	0.478	-0.367	0.178	0.478
G830V	0.211	0.444	0.989	0.211
831	1.111	0.178	0.722	1.111
832	1.656	1.078	1.622	1.622
833	1.378	1.622	2.167	1.344
834	0.489	1.344	1.889	0.456
835	0.144	0.144	1.000	0.111

I836V	0.733	0.733	0.733	0.700
837	1.433	1.433	1.433	1.400
838	0.622	0.622	0.622	0.589
839	0.844	0.844	0.844	0.811
840	0.333	0.333	0.333	0.300
841	-0.244	-0.244	-0.244	-0.244
In the region 842-843 there are no changes				
844	1.567	1.567	1.567	1.567
845	1.567	1.278	1.567	1.567
846	1.278	0.989	1.278	1.278
847	1.978	1.689	1.978	1.978
848	2.056	1.767	2.056	2.056
I849M	1.956	1.667	1.711	1.956
850	1.989	1.700	1.744	1.989
851	1.933	1.644	1.689	1.933
852	1.044	0.756	0.800	1.044
Y853N	0.967	0.678	0.722	0.967
854	0.389	0.389	0.144	0.389
855	0.900	0.900	0.656	0.900
856	1.011	1.011	0.767	1.011
857	0.933	0.933	0.689	0.933
858	1.500	1.500	1.500	1.500
In the region 859-887 there are no changes				
888	-1.667	-1.667	-1.667	-1.667
889	-2.178	-1.889	-2.167	-2.178
890	-1.789	-1.500	-1.778	-1.789
891	-1.833	-1.544	-1.822	-1.833
892	-0.900	-0.611	-0.889	-0.900
S893A/T	-0.267	0.022	-0.256	-0.267

894	0.044	0.333	0.056	0.044
895	-0.767	-0.478	-0.756	-0.767
896	-0.178	0.111	-0.167	-0.178
897	0.367	0.656	0.378	0.367
898	0.067	0.067	0.067	0.067
In the region 890-901 there are no changes				
902	-0.811	-0.811	-0.811	-0.811
903	-0.267	-0.422	-0.267	-0.267
904	0.589	0.433	0.589	0.589
905	0.300	0.144	0.300	0.300
906	0.222	0.067	0.222	0.222
V907F	0.533	0.378	0.533	0.533
908	0.533	0.378	0.533	0.533
909	-0.167	-0.322	-0.167	-0.167
910	-0.056	-0.211	-0.056	-0.056
911	-0.411	-0.567	-0.411	-0.411
912	-0.567	-0.567	-0.567	-0.567
In the region 913-922 there are no changes				
923	0.111	0.111	0.111	0.111
924	-0.244	-0.189	-0.244	-0.244
925	-0.600	-0.544	-0.600	-0.600
926	-1.189	-1.133	-1.189	-1.189
927	-0.889	-0.833	-0.889	-0.889
Y928S	-0.378	-0.322	-0.378	-0.378
929	-1.300	-1.244	-1.300	-1.300
930	-1.344	-1.289	-1.344	-1.344
931	-1.611	-1.556	-1.611	-1.611
932	-1.789	-1.733	-1.789	-1.789
933	-1.144	-1.144	-1.144	-1.144

In the region 934-972 there are no changes				
973	1.433	1.433	1.433	1.433
974	1.533	1.422	1.533	1.533
975	1.533	1.422	1.533	1.533
976	1.422	1.311	1.044	1.422
977	2.278	2.167	1.900	2.278
F978A	3.133	3.022	2.756	3.133
979	2.978	2.867	2.600	2.978
A980P	2.511	2.400	2.133	2.511
981	2.289	2.178	1.911	2.289
982	2.033	1.922	1.656	2.289
983	1.922	1.922	1.544	2.178
984	2.478	2.478	2.100	2.733
985	2.233	2.233	2.233	2.489
M986V	1.378	1.378	1.378	1.633
987	1.378	1.378	1.378	1.633
988	0.978	0.978	0.978	1.233
989	0.933	0.933	0.933	1.189
990	1.044	1.044	1.044	1.300
991	1.033	1.033	1.033	1.033
In the region 992-993 there are no changes				
994	-0.300	-0.300	-0.300	-0.300
995	0.289	0.011	0.289	0.289
996	-0.611	-0.889	-0.611	-0.611
997	-0.322	-0.600	-0.322	-0.322
998	-0.667	-0.944	-0.667	-0.667
A999T	-0.478	-0.756	-0.478	-0.478
1000	-0.767	-1.044	-0.767	-0.767
1001	-0.389	-0.667	-0.389	-0.389

1002	0.200	-0.078	0.200	0.200
1003	-0.011	-0.289	-0.011	-0.011
1004	0.289	0.289	0.289	0.289
In the region 1005-1045 there are no changes				
1046	-1.044	-1.044	-1.044	-1.044
1047	-1.533	-1.156	-1.533	-1.533
1048	-0.678	-0.300	-0.678	-0.678
1049	-0.111	0.267	-0.111	-0.111
1050	-0.322	0.056	-0.322	-0.322
P1051A	-0.289	0.089	-0.289	-0.289
1052	0.633	1.011	0.633	0.633
1053	0.722	1.100	0.722	0.722
1054	1.533	1.911	1.533	1.533
1055	0.644	1.022	0.644	0.644
1056	1.289	1.289	1.289	1.289
In the region 1057-1101 there are no changes				
1102	-0.800	-0.800	-0.800	-0.800
1103	-1.144	-0.933	-1.144	-1.144
1104	-0.811	-0.600	-1.211	-0.811
1105	0.000	0.211	-0.400	0.000
1106	-1.000	-0.789	-1.400	-1.000
Q1107P	-0.367	-0.156	-0.767	-0.367
W1108R	-0.222	-0.011	-0.622	-0.222
1109	-0.222	-0.011	-0.622	-0.222
1110	0.122	0.333	-0.278	0.122
1111	0.156	0.367	-0.244	0.156
1112	1.011	1.011	0.611	1.011
1113	1.022	1.022	1.022	1.022
In the region 1114-1135 there are no changes				

1136	-0.422	-0.422	-0.422	-0.422
1137	-0.711	-0.700	-0.711	-0.711
1138	-0.956	-0.944	-0.956	-0.956
1139	-1.300	-1.289	-1.300	-1.300
1140	-1.300	-1.289	-1.300	-1.300
S1141T	-0.411	-0.400	-0.411	-0.411
1142	0.144	0.156	0.144	0.144
1143	0.144	0.156	0.144	0.144
1144	-0.122	-0.111	-0.122	-0.122
1145	-0.389	-0.378	-0.389	-0.389
1146	-0.733	-0.733	-0.733	-0.733
In the region 1147-1245 there are no changes				
1246	0.844	0.844	0.844	0.844
1247	0.889	0.922	0.889	0.889
1248	-0.044	-0.011	-0.044	-0.044
1249	-0.900	-0.867	-0.900	-0.900
1250	-1.722	-1.689	-1.722	-1.722
V1251I	-2.078	-2.044	-2.078	-2.078
1252	-1.767	-1.733	-2.122	-1.767
1253	-1.733	-1.700	-2.089	-1.733
1254	-2.078	-2.044	-2.433	-2.078
1255	-1.967	-1.933	-2.322	-1.967
T1256K	-2.011	-2.011	-2.367	-2.011
1257	-1.156	-1.156	-1.511	-1.156
1258	-0.567	-0.567	-0.922	-0.567
1259	-0.600	-0.600	-0.956	-0.600
1260	-0.989	-0.989	-1.344	-0.989
1261	-0.956	-0.956	-0.956	-0.956
In the region 1262-1276 there are no changes				

Table S6. Changes in local flexibility resulting from amino acid substitutions. Flexibility values were calculated using the ProtScale tool with a nine-residue sliding window. The window centered on the substituted residue is highlighted in light orange for the wild-type protein and in blue for the mutant proteins. Regions unaffected by the substitutions are omitted.

Aminoacid number and mutation	Flexibility values for 9 aminoacids windows for wild-type protein	Flexibility values for allelic sequence with N15D, N44S, H61Y, A80E, M89T, F103L, Y116C, N183S, I261V, L305P, S400N, R492C, E566K, R580P, R593C, L662R, C717Y, S795C, I829V, I849M, S893A, V907F, Y928S, F978A, A999T, P1051A, Q1107P, S1141T and V1251I	Flexibility values for allelic sequence with F17L, A80G, G185V, A599T, R669C, V801M, G830V, Y853N, S893T, A980P, W1108R and T1256K	Flexibility values for allelic sequence with N21D, I186N, I836V and M986V
In the region 5-9 there are no changes				
10	0.483	0.483	0.483	0.483
11	0.478	0.483	0.478	0.478
12	0.453	0.459	0.453	0.453
13	0.437	0.442	0.443	0.437
14	0.429	0.434	0.436	0.429
N15D	0.410	0.416	0.417	0.410
16	0.421	0.427	0.428	0.421
F17L	0.420	0.426	0.427	0.426
18	0.420	0.426	0.427	0.426
19	0.424	0.430	0.431	0.430
20	0.429	0.429	0.436	0.434
N21D	0.447	0.447	0.453	0.452
22	0.469	0.469	0.469	0.474
23	0.469	0.469	0.469	0.474
24	0.480	0.480	0.480	0.486
25	0.484	0.484	0.484	0.490
26	0.486	0.486	0.486	0.486
In the region 27-38 there are no changes				
39	0.421	0.421	0.421	0.421
40	0.416	0.421	0.416	0.416

41	0.407	0.412	0.407	0.407
42	0.413	0.419	0.413	0.413
43	0.413	0.419	0.413	0.413
N44S	0.432	0.438	0.432	0.432
45	0.439	0.444	0.439	0.439
46	0.427	0.432	0.427	0.427
47	0.413	0.419	0.413	0.413
48	0.400	0.406	0.400	0.400
49	0.392	0.392	0.392	0.392
In the region 50-55 there are no changes				
56	0.419	0.419	0.419	0.419
57	0.411	0.422	0.411	0.411
58	0.428	0.439	0.428	0.428
59	0.408	0.419	0.408	0.408
60	0.419	0.430	0.419	0.419
H61Y	0.419	0.430	0.419	0.419
62	0.436	0.447	0.436	0.436
63	0.437	0.448	0.437	0.437
64	0.419	0.430	0.419	0.419
65	0.401	0.412	0.401	0.401
66	0.407	0.407	0.407	0.407
In the region 67-74 there are no changes				
75	0.418	0.418	0.418	0.418
76	0.414	0.430	0.434	0.414
77	0.431	0.447	0.451	0.431
78	0.411	0.427	0.431	0.411
79	0.416	0.431	0.436	0.416
A80E/G	0.433	0.449	0.453	0.433
81	0.426	0.441	0.446	0.426

82	0.424	0.440	0.444	0.424
83	0.430	0.446	0.450	0.430
84	0.437	0.452	0.457	0.437
85	0.430	0.446	0.430	0.430
86	0.436	0.451	0.436	0.436
87	0.447	0.462	0.447	0.447
88	0.438	0.453	0.438	0.438
M89T	0.436	0.451	0.436	0.436
90	0.446	0.461	0.446	0.446
91	0.449	0.464	0.449	0.449
92	0.449	0.464	0.449	0.449
93	0.464	0.480	0.464	0.464
94	0.482	0.482	0.482	0.482
In the region 95-97 there are no changes				
98	0.491	0.491	0.491	0.491
99	0.474	0.481	0.474	0.474
100	0.450	0.457	0.450	0.450
101	0.427	0.433	0.427	0.427
102	0.421	0.428	0.421	0.421
F103L	0.411	0.418	0.411	0.411
104	0.416	0.422	0.416	0.416
105	0.414	0.421	0.414	0.414
106	0.422	0.429	0.422	0.422
107	0.396	0.402	0.396	0.396
108	0.410	0.410	0.410	0.410
In the region 109-110 there are no changes				
111	0.437	0.437	0.437	0.437
112	0.442	0.434	0.442	0.442
113	0.433	0.426	0.433	0.433

114	0.424	0.417	0.424	0.424
115	0.424	0.417	0.424	0.424
Y116C	0.451	0.443	0.451	0.451
117	0.453	0.446	0.453	0.453
118	0.454	0.447	0.454	0.454
119	0.448	0.440	0.448	0.448
120	0.468	0.460	0.468	0.468
121	0.464	0.464	0.464	0.464
In the region 122-177 there are no changes				
178	0.466	0.466	0.466	0.466
179	0.458	0.463	0.458	0.458
180	0.472	0.478	0.472	0.472
181	0.483	0.489	0.467	0.483
182	0.478	0.483	0.461	0.478
N183S	0.481	0.487	0.464	0.481
184	0.494	0.500	0.478	0.494
G185V	0.490	0.496	0.473	0.490
I186N	0.489	0.494	0.472	0.489
187	0.498	0.503	0.481	0.498
188	0.480	0.480	0.463	0.480
189	0.459	0.459	0.442	0.459
190	0.433	0.433	0.433	0.433
191	0.437	0.437	0.437	0.437
In the region 192-255 there are no changes				
256	0.399	0.399	0.399	0.399
257	0.410	0.402	0.410	0.410
258	0.426	0.418	0.426	0.426
259	0.434	0.427	0.434	0.434
260	0.422	0.414	0.422	0.422

I261V	0.418	0.410	0.418	0.418
262	0.414	0.407	0.414	0.414
263	0.408	0.400	0.408	0.408
264	0.428	0.420	0.428	0.428
265	0.448	0.440	0.448	0.448
266	0.451	0.451	0.451	0.451
In the region 267-299 there are no changes				
300	0.426	0.426	0.426	0.426
301	0.416	0.431	0.416	0.416
302	0.416	0.431	0.416	0.416
303	0.406	0.421	0.406	0.406
304	0.394	0.410	0.394	0.394
L305P	0.391	0.407	0.391	0.391
306	0.398	0.413	0.398	0.398
307	0.398	0.413	0.398	0.398
308	0.404	0.420	0.404	0.404
309	0.403	0.419	0.403	0.403
310	0.397	0.397	0.397	0.397
In the region 309-394 there are no changes				
395	0.406	0.406	0.406	0.406
396	0.411	0.406	0.411	0.411
397	0.417	0.411	0.417	0.417
398	0.418	0.412	0.418	0.418
399	0.440	0.434	0.440	0.440
S400N	0.440	0.434	0.440	0.440
401	0.441	0.436	0.441	0.441
402	0.453	0.448	0.453	0.453
403	0.461	0.456	0.461	0.461
404	0.479	0.473	0.479	0.479

405	0.473	0.473	0.473	0.473
In the region 404-486 there are no changes				
487	0.463	0.463	0.463	0.463
488	0.473	0.453	0.473	0.473
489	0.478	0.458	0.478	0.478
490	0.489	0.469	0.489	0.489
491	0.477	0.457	0.477	0.477
R492C	0.474	0.454	0.474	0.474
493	0.457	0.437	0.457	0.457
494	0.454	0.434	0.454	0.454
495	0.463	0.443	0.463	0.463
496	0.454	0.434	0.454	0.454
497	0.451	0.451	0.451	0.451
In the region 498-560 there are no changes				
561	0.444	0.444	0.444	0.444
562	0.460	0.457	0.460	0.460
563	0.451	0.448	0.451	0.451
564	0.438	0.434	0.438	0.438
565	0.441	0.438	0.441	0.441
E566K	0.454	0.451	0.454	0.454
567	0.441	0.438	0.441	0.441
568	0.432	0.429	0.432	0.432
569	0.418	0.414	0.418	0.418
570	0.418	0.414	0.418	0.418
571	0.414	0.414	0.414	0.414
In the region 572-574 there are no changes				
575	0.444	0.444	0.444	0.444
576	0.460	0.458	0.460	0.460
577	0.469	0.467	0.469	0.469

578	0.477	0.474	0.477	0.477
579	0.471	0.469	0.471	0.471
R580P	0.462	0.460	0.462	0.462
581	0.473	0.471	0.473	0.473
582	0.454	0.452	0.454	0.454
583	0.438	0.436	0.438	0.438
584	0.437	0.434	0.437	0.437
585	0.419	0.419	0.419	0.419
In the region 586-587 there are no changes				
588	0.419	0.419	0.419	0.419
589	0.434	0.414	0.434	0.434
590	0.434	0.414	0.434	0.434
591	0.434	0.414	0.434	0.434
592	0.456	0.436	0.456	0.456
R593C	0.440	0.420	0.440	0.440
594	0.450	0.430	0.450	0.450
595	0.433	0.413	0.442	0.433
596	0.444	0.424	0.453	0.444
597	0.436	0.416	0.444	0.436
598	0.433	0.433	0.442	0.433
A599T	0.439	0.439	0.448	0.439
600	0.459	0.459	0.468	0.459
601	0.446	0.446	0.454	0.446
602	0.453	0.453	0.462	0.453
603	0.446	0.446	0.454	0.446
604	0.461	0.461	0.461	0.461
In the region 605-656 there are no changes				
657	0.483	0.483	0.483	0.483
658	0.491	0.509	0.491	0.491

659	0.486	0.503	0.486	0.486
660	0.488	0.506	0.488	0.488
661	0.489	0.507	0.489	0.489
L662R	0.491	0.509	0.491	0.491
663	0.491	0.509	0.491	0.491
664	0.481	0.499	0.481	0.481
665	0.483	0.501	0.463	0.483
666	0.486	0.503	0.466	0.486
667	0.501	0.501	0.481	0.501
668	0.493	0.493	0.473	0.493
R669C	0.493	0.493	0.473	0.493
670	0.501	0.501	0.481	0.501
671	0.499	0.499	0.479	0.499
672	0.497	0.497	0.477	0.497
673	0.488	0.488	0.468	0.488
674	0.483	0.483	0.483	0.483
In the region 675-711 there are no changes				
712	0.397	0.397	0.397	0.397
713	0.401	0.409	0.401	0.401
714	0.384	0.392	0.384	0.384
715	0.389	0.397	0.389	0.389
716	0.406	0.413	0.406	0.406
C717Y	0.413	0.421	0.413	0.413
718	0.430	0.438	0.430	0.430
719	0.430	0.438	0.430	0.430
720	0.428	0.436	0.428	0.428
721	0.448	0.456	0.448	0.448
722	0.466	0.466	0.466	0.466
In the region 723-789 there are no changes				

790	0.428	0.428	0.428	0.428
791	0.432	0.414	0.432	0.432
792	0.407	0.389	0.407	0.407
793	0.407	0.389	0.407	0.407
794	0.407	0.389	0.407	0.407
S795C	0.414	0.397	0.414	0.414
796	0.438	0.420	0.438	0.438
797	0.438	0.420	0.428	0.438
798	0.460	0.442	0.450	0.460
799	0.436	0.418	0.426	0.436
800	0.413	0.413	0.403	0.413
V801M	0.437	0.437	0.427	0.437
802	0.452	0.452	0.442	0.452
803	0.450	0.450	0.440	0.450
804	0.448	0.448	0.438	0.448
805	0.442	0.442	0.432	0.442
806	0.448	0.448	0.448	0.448
In the region 807-823 there are no changes				
824	0.438	0.438	0.438	0.438
825	0.438	0.430	0.438	0.438
826	0.441	0.433	0.424	0.441
827	0.458	0.450	0.441	0.458
828	0.477	0.469	0.460	0.477
I829V	0.463	0.456	0.447	0.463
G830V	0.460	0.452	0.443	0.460
831	0.451	0.443	0.434	0.451
832	0.442	0.434	0.426	0.434
833	0.451	0.443	0.434	0.443
834	0.454	0.454	0.438	0.447

835	0.446	0.446	0.446	0.438
I836V	0.440	0.440	0.440	0.432
837	0.421	0.421	0.421	0.413
838	0.431	0.431	0.431	0.423
839	0.432	0.432	0.432	0.424
840	0.449	0.449	0.449	0.441
841	0.447	0.447	0.447	0.447
In the region 842-843 there are no changes				
844	0.454	0.454	0.454	0.454
845	0.454	0.437	0.454	0.454
846	0.471	0.453	0.471	0.471
847	0.454	0.437	0.454	0.454
848	0.464	0.447	0.464	0.464
I849M	0.451	0.433	0.456	0.451
850	0.462	0.444	0.467	0.462
851	0.437	0.419	0.441	0.437
852	0.440	0.422	0.444	0.440
Y853N	0.430	0.412	0.434	0.430
854	0.428	0.428	0.432	0.428
855	0.412	0.412	0.417	0.412
856	0.419	0.419	0.423	0.419
857	0.409	0.409	0.413	0.409
858	0.403	0.403	0.403	0.403
In the region 859-887 there are no changes				
888	0.467	0.467	0.467	0.467
889	0.482	0.466	0.474	0.482
890	0.490	0.473	0.482	0.490
891	0.486	0.469	0.478	0.486
892	0.484	0.468	0.477	0.484

S893A/T	0.472	0.456	0.464	0.472
894	0.466	0.449	0.458	0.466
895	0.480	0.463	0.472	0.480
896	0.464	0.448	0.457	0.464
897	0.456	0.439	0.448	0.456
898	0.454	0.454	0.454	0.454
In the region 899-901 there are no changes				
902	0.444	0.444	0.444	0.444
903	0.439	0.430	0.439	0.439
904	0.427	0.418	0.427	0.427
905	0.443	0.434	0.443	0.443
906	0.433	0.424	0.433	0.433
V907F	0.427	0.418	0.427	0.427
908	0.430	0.421	0.430	0.430
909	0.451	0.442	0.451	0.451
910	0.447	0.438	0.447	0.447
911	0.450	0.441	0.450	0.450
912	0.441	0.441	0.441	0.441
In the region 911-922 there are no changes				
923	0.427	0.427	0.427	0.427
924	0.440	0.450	0.440	0.440
925	0.452	0.462	0.452	0.452
926	0.463	0.473	0.463	0.463
927	0.466	0.476	0.466	0.466
Y928S	0.450	0.460	0.450	0.450
929	0.468	0.478	0.468	0.468
930	0.466	0.476	0.466	0.466
931	0.462	0.472	0.462	0.462
932	0.441	0.451	0.441	0.441

933	0.446	0.446	0.446	0.446
In the region 934-972 there are no changes				
973	0.406	0.406	0.406	0.406
974	0.407	0.412	0.407	0.407
975	0.407	0.412	0.407	0.407
976	0.412	0.418	0.429	0.412
977	0.400	0.406	0.417	0.400
F978A	0.387	0.392	0.403	0.387
979	0.378	0.383	0.394	0.378
A980P	0.397	0.402	0.413	0.397
981	0.396	0.401	0.412	0.396
982	0.386	0.391	0.402	0.396
983	0.391	0.391	0.408	0.401
984	0.378	0.378	0.394	0.388
985	0.398	0.398	0.398	0.408
M986V	0.409	0.409	0.409	0.419
987	0.409	0.409	0.409	0.419
988	0.431	0.431	0.431	0.441
989	0.428	0.428	0.428	0.438
990	0.422	0.422	0.422	0.432
991	0.429	0.429	0.429	0.429
In the region 992-993 there are no changes				
994	0.446	0.446	0.446	0.446
995	0.431	0.440	0.431	0.431
996	0.440	0.449	0.440	0.440
997	0.423	0.432	0.423	0.423
998	0.419	0.428	0.419	0.419
A999T	0.436	0.444	0.436	0.436
1000	0.452	0.461	0.452	0.452

1001	0.436	0.444	0.436	0.436
1002	0.419	0.428	0.419	0.419
1003	0.408	0.417	0.408	0.408
1004	0.419	0.419	0.419	0.419
In the region 1005-1045 there are no changes				
1046	0.461	0.461	0.461	0.461
1047	0.483	0.467	0.483	0.483
1048	0.476	0.459	0.476	0.476
1049	0.470	0.453	0.470	0.470
1050	0.468	0.451	0.468	0.468
P1051A	0.479	0.462	0.479	0.479
1052	0.461	0.444	0.461	0.461
1053	0.461	0.444	0.461	0.461
1054	0.446	0.429	0.446	0.446
1055	0.450	0.433	0.450	0.450
1056	0.437	0.437	0.437	0.437
In the region 1057-1101 there are no changes				
1102	0.466	0.466	0.466	0.466
1103	0.460	0.462	0.460	0.460
1104	0.442	0.444	0.467	0.442
1105	0.428	0.430	0.452	0.428
1106	0.436	0.438	0.460	0.436
Q1107P	0.423	0.426	0.448	0.423
W1108R	0.400	0.402	0.424	0.400
1109	0.400	0.402	0.424	0.400
1110	0.409	0.411	0.433	0.409
1111	0.417	0.419	0.441	0.417
1112	0.406	0.406	0.430	0.406
1113	0.428	0.428	0.428	0.428

In the region 1114-1135 there are no changes				
1136	0.457	0.457	0.457	0.457
1137	0.473	0.466	0.473	0.473
1138	0.481	0.473	0.481	0.481
1139	0.477	0.469	0.477	0.477
1140	0.476	0.468	0.476	0.476
S1141T	0.476	0.468	0.476	0.476
1142	0.462	0.454	0.462	0.462
1143	0.462	0.454	0.462	0.462
1144	0.459	0.451	0.459	0.459
1145	0.456	0.448	0.456	0.456
1146	0.451	0.451	0.451	0.451
In the region 1147-1245 there are no changes				
1246	0.438	0.438	0.438	0.438
1247	0.440	0.448	0.440	0.440
1248	0.441	0.449	0.441	0.441
1249	0.453	0.461	0.453	0.453
1250	0.446	0.453	0.446	0.446
V1251I	0.471	0.479	0.471	0.471
1252	0.466	0.473	0.469	0.466
1253	0.450	0.458	0.453	0.450
1254	0.444	0.452	0.448	0.444
1255	0.440	0.448	0.443	0.440
T1256K	0.438	0.438	0.441	0.438
1257	0.427	0.427	0.430	0.427
1258	0.411	0.411	0.414	0.411
1259	0.430	0.430	0.433	0.430
1260	0.422	0.422	0.426	0.422
1261	0.433	0.433	0.433	0.433

In the region 1262-1276 there are no changes

Table S7. Superimposition of native and mutant protein structures highlighting a nine-residue region centered on the substituted amino acid (the native protein is shown in brown and the mutant protein in blue) and comparison of local electrostatic potential and hydrophobicity distributions between native and mutant P-gp. Hydrophobic regions are shown in orange, whereas hydrophilic regions are shown in blue. For electrostatic potential mapping, negatively charged regions are colored red and positively charged regions are colored blue.

Superimposition of native and mutant protein illustrated only for a window of 9 amino-acid centered on the mutated residue and RMSD value	distribution of electrostatic potential		distribution of hydrophobicity	
	native protein	mutant protein	native protein	mutant protein
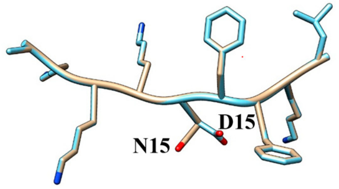 0.049 Å	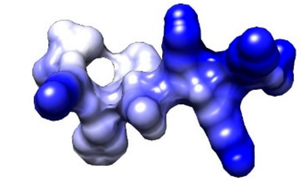	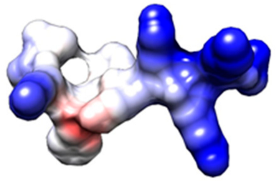	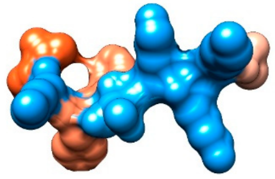	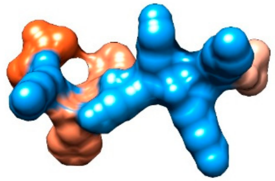
		N15D		
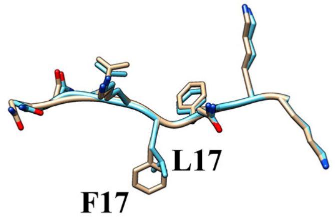 0.198 Å	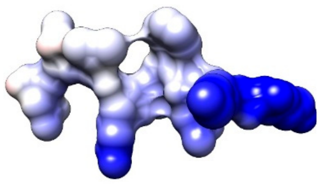	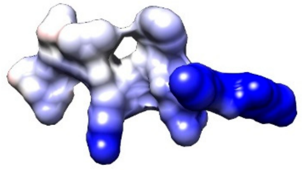	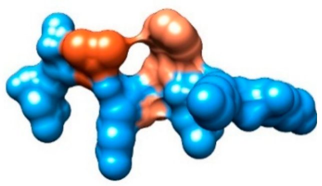	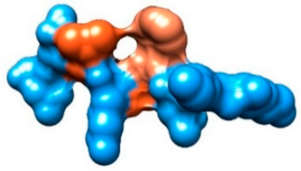
		F17L		
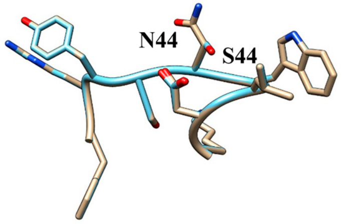 0.031 Å	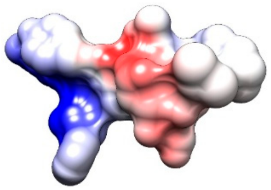	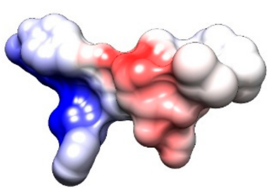	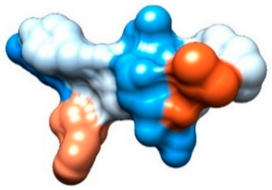	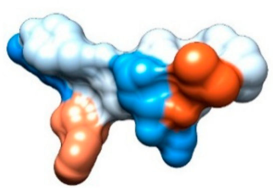
		N44S		

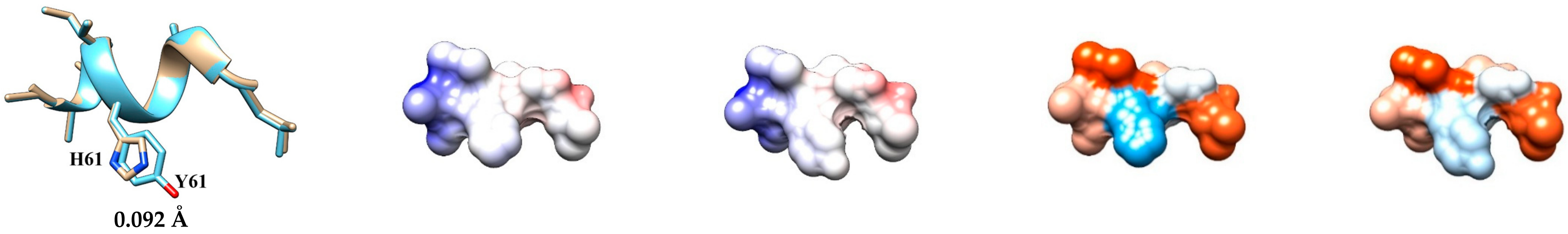

H61Y

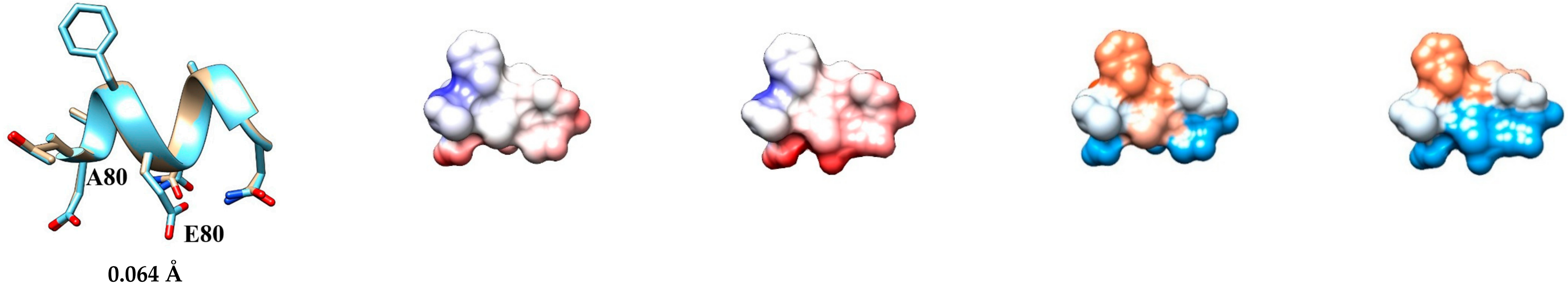

A80E

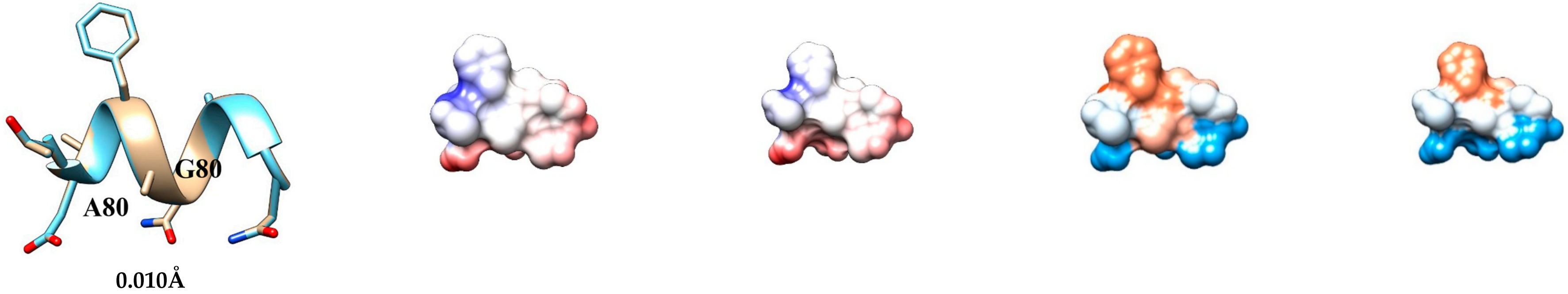

A80G

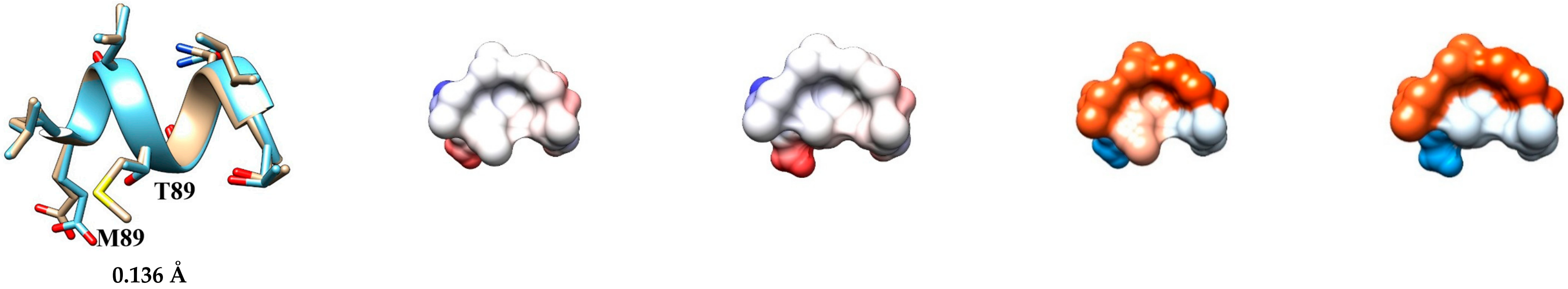

M89T

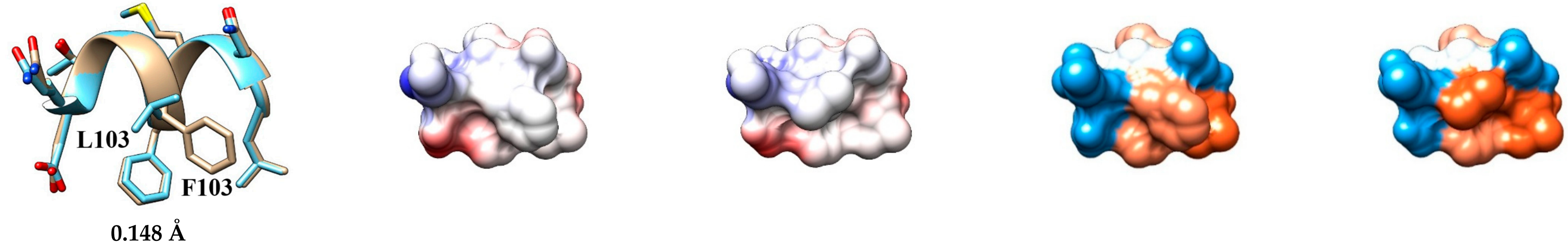

F103L

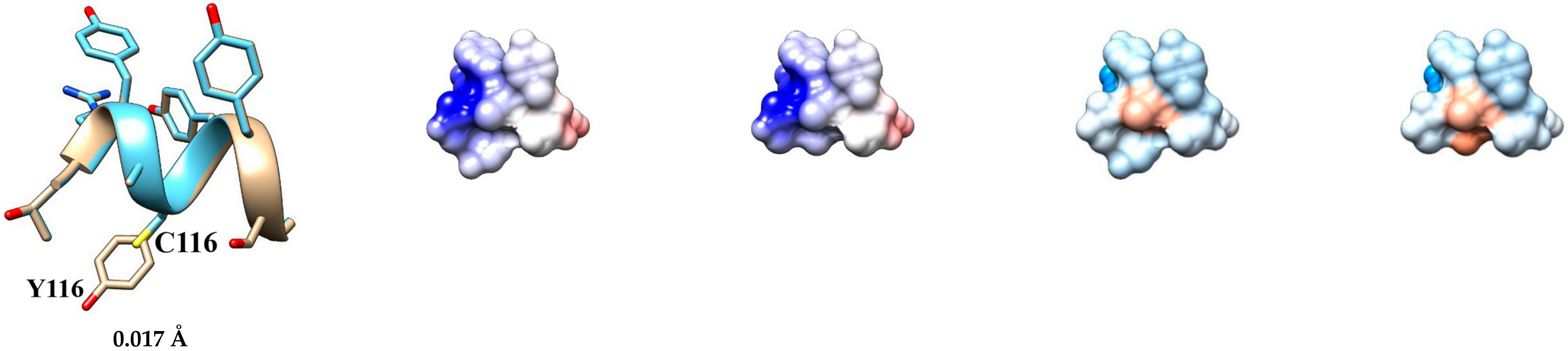

Y116C

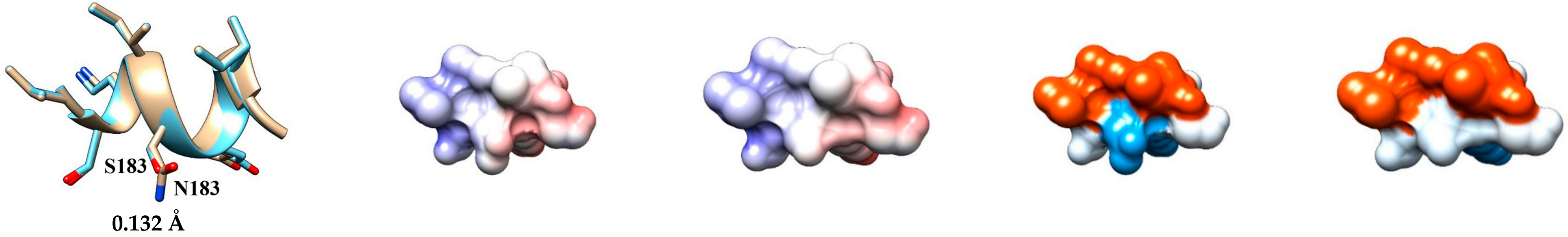

N183S

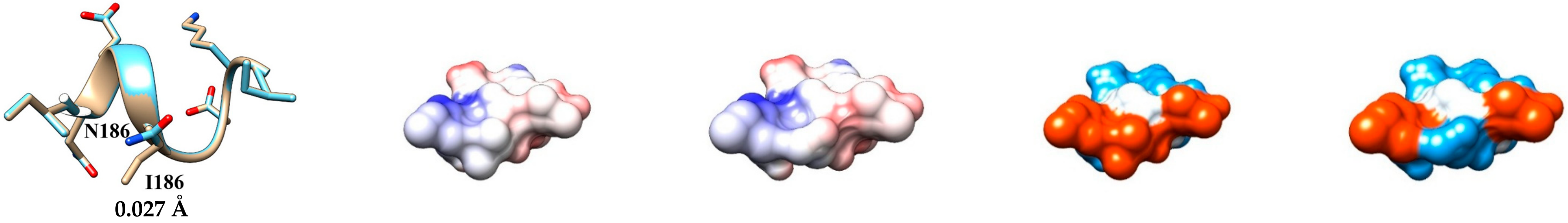

I186N

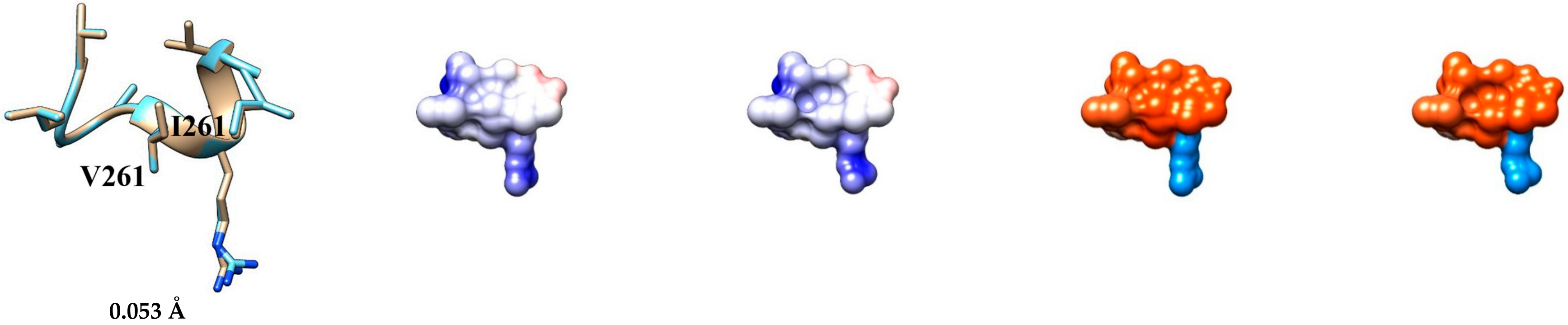

I261N

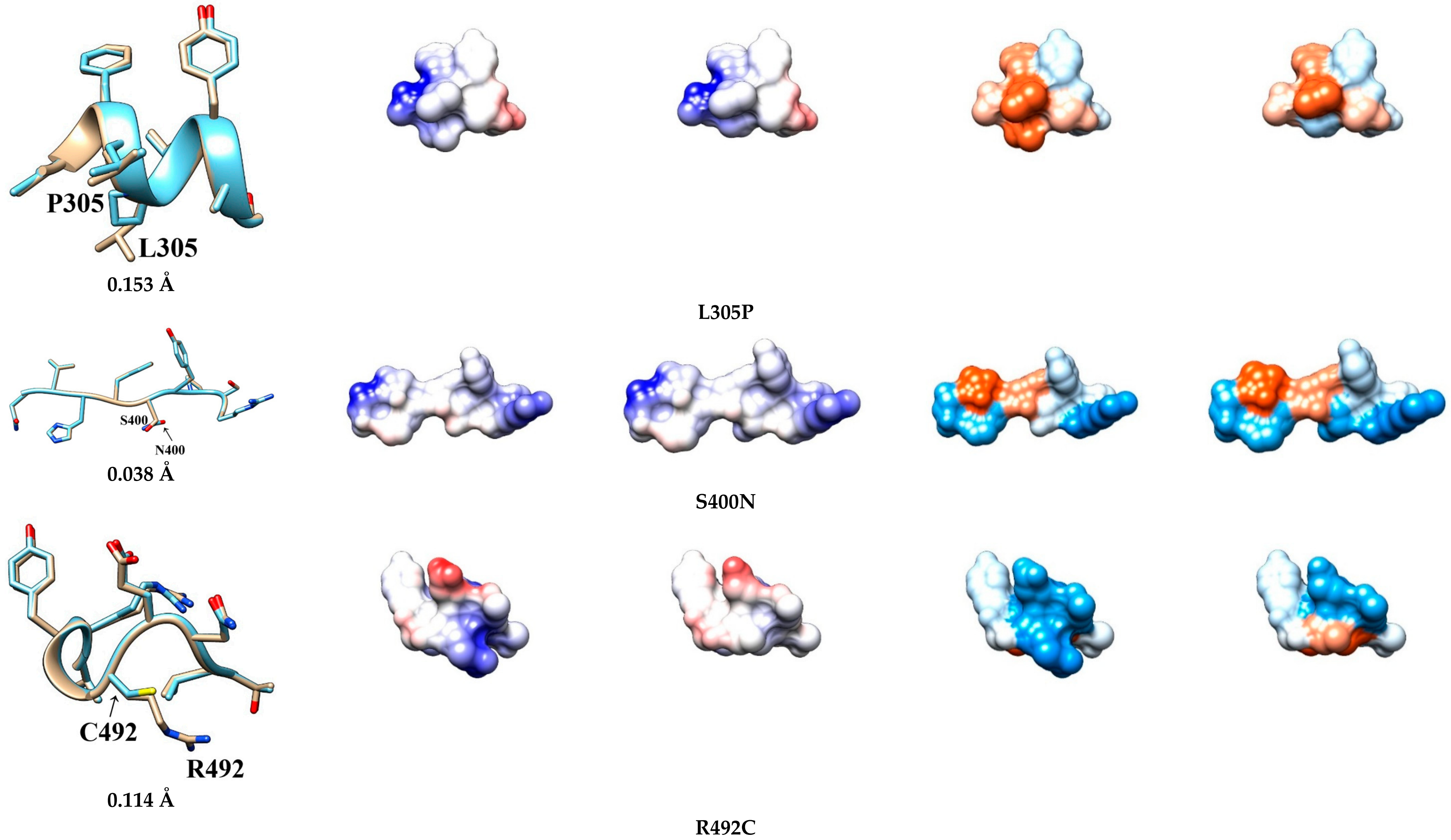
P305
L305
0.153 Å
L305P
S400
N400
0.038 Å
S400N
C492
R492
0.114 Å
R492C

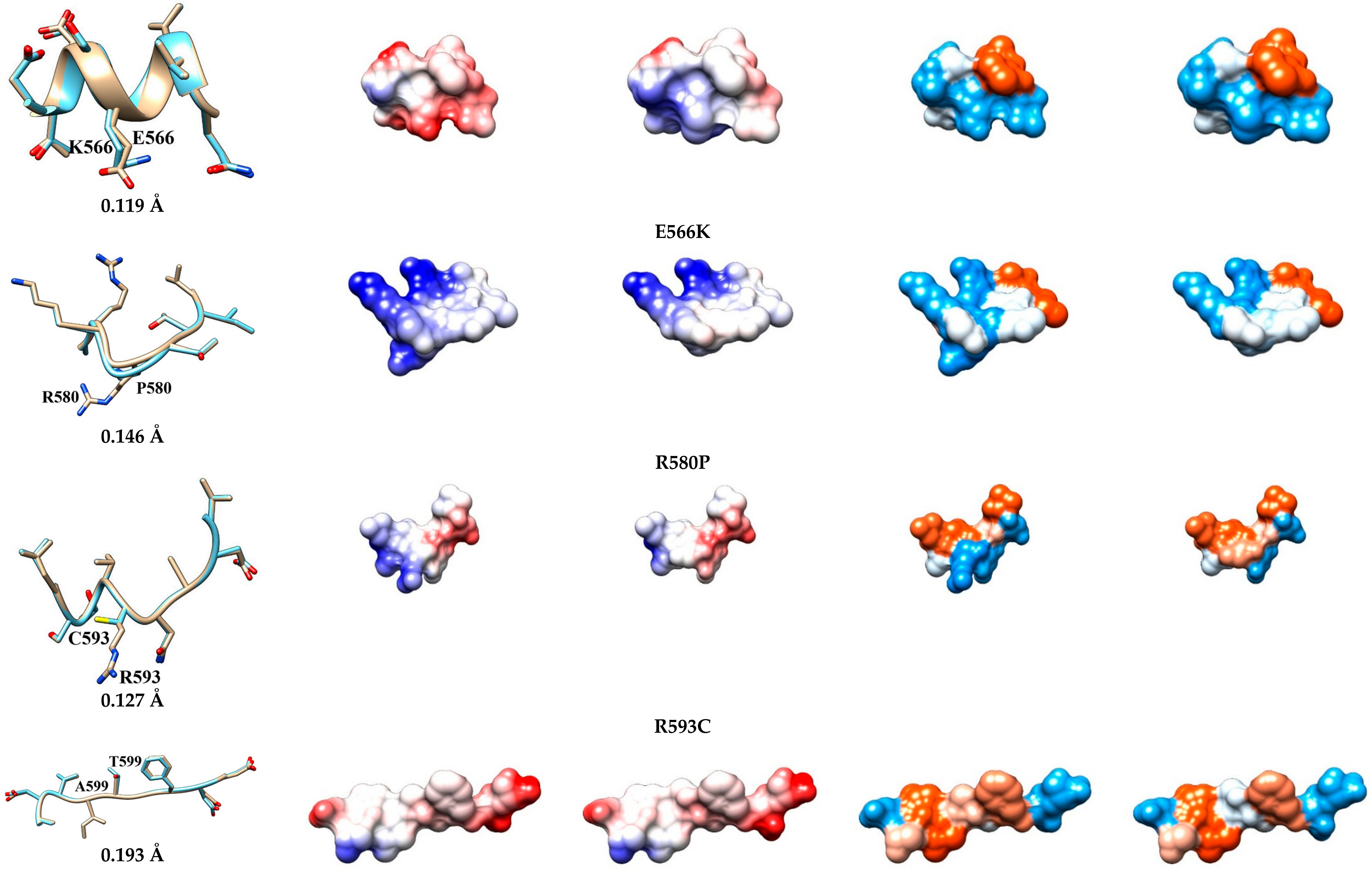
K566
E566
0.119 Å
E566K
R580
P580
0.146 Å
R580P
C593
R593
0.127 Å
R593C
T599
A599
0.193 Å

A599T

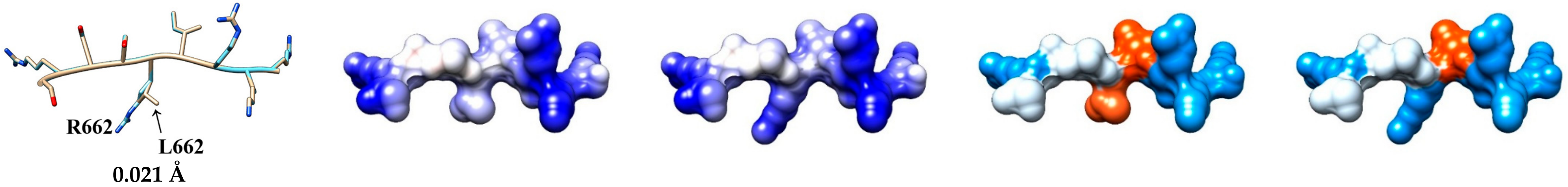

L662R

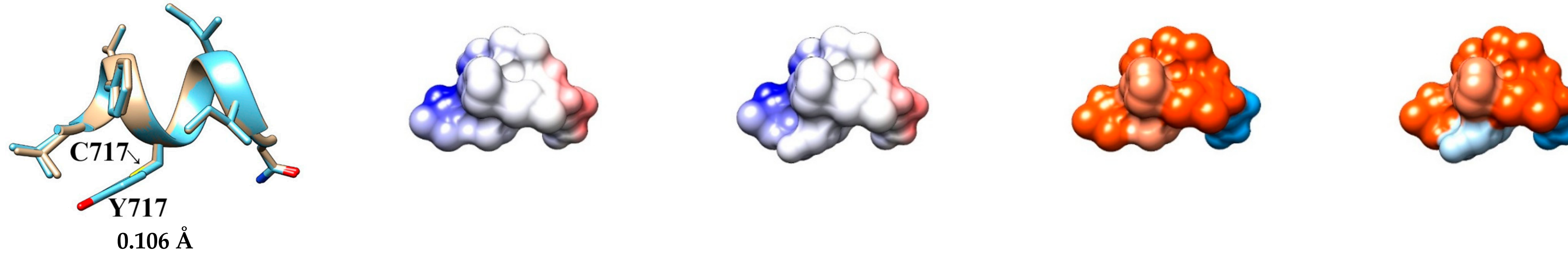

C717Y

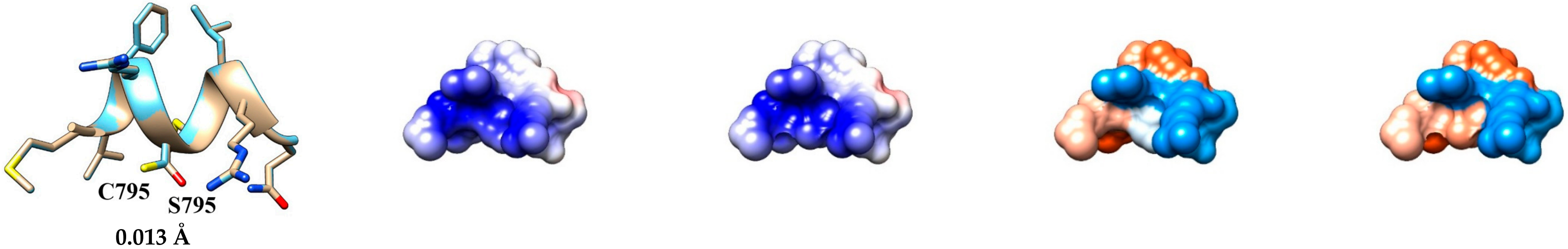

S795C

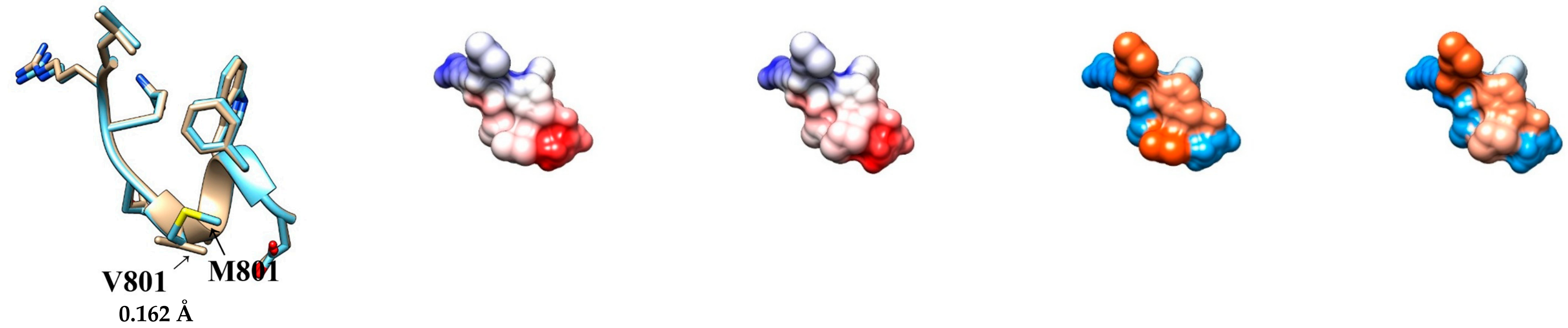

V801M

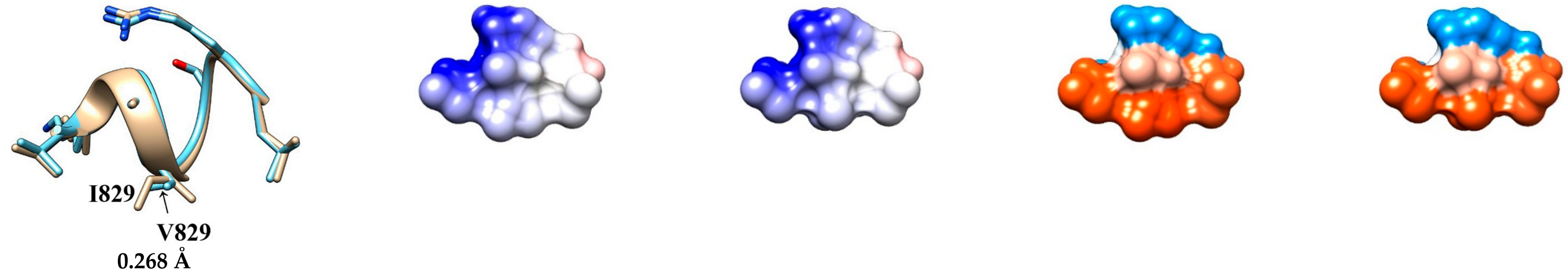

I829V

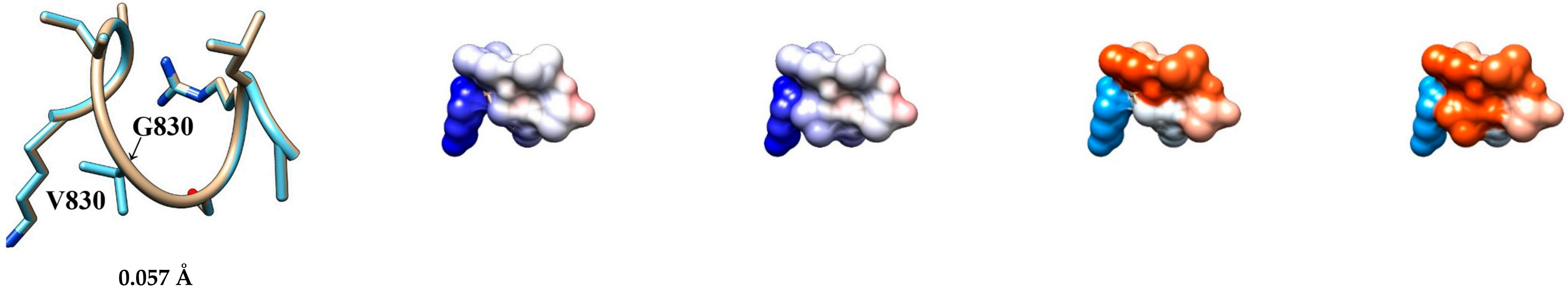

G830V

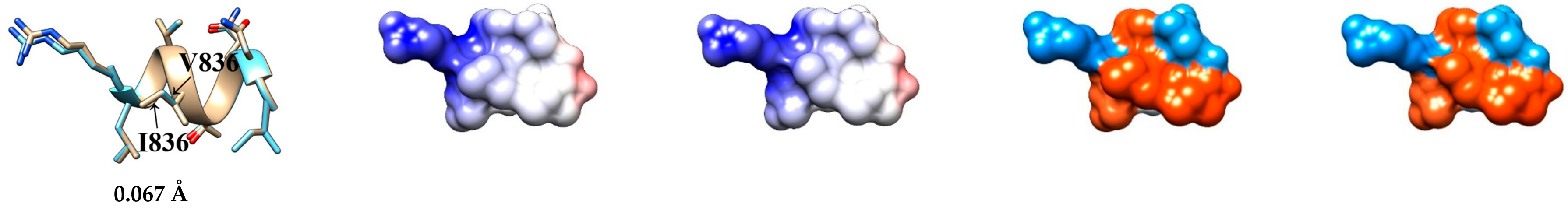

I836V

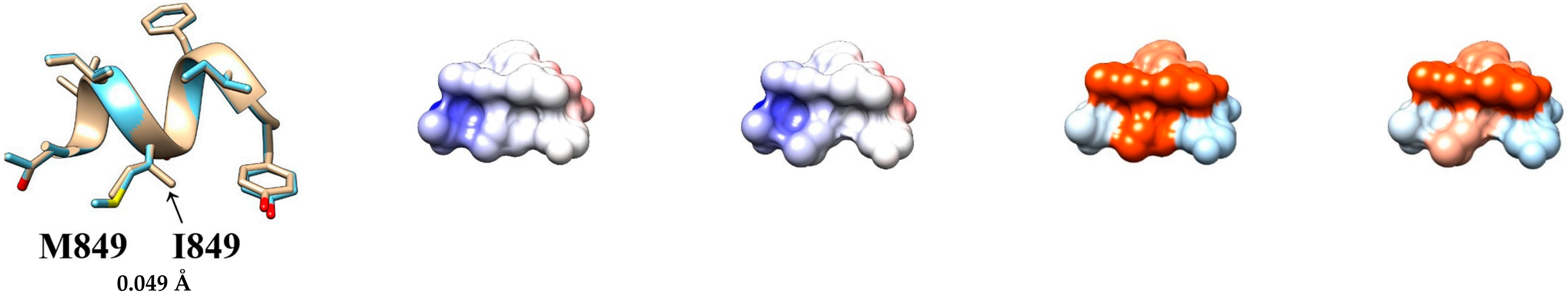

I849M

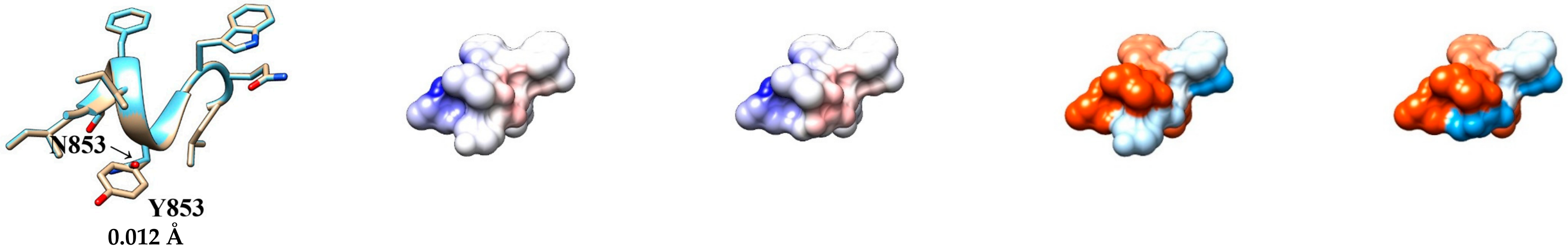

Y853N

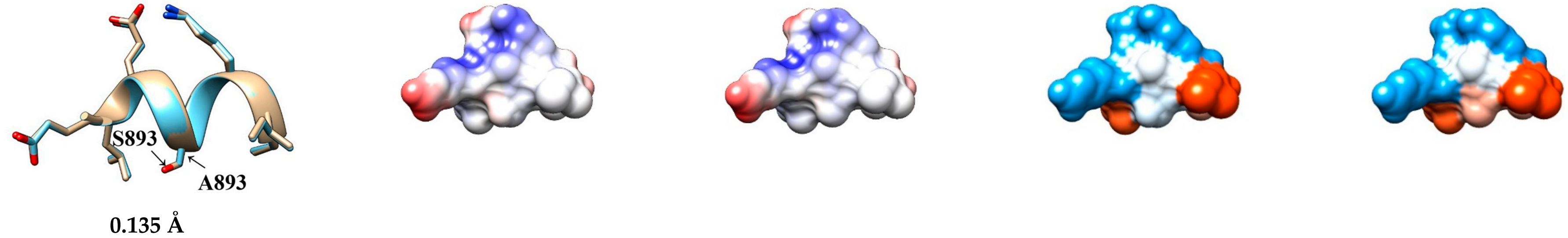

S893A

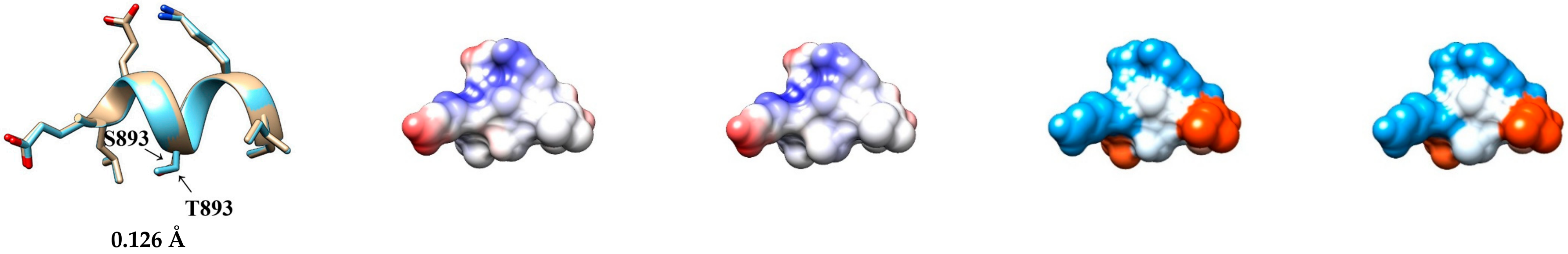

S893T

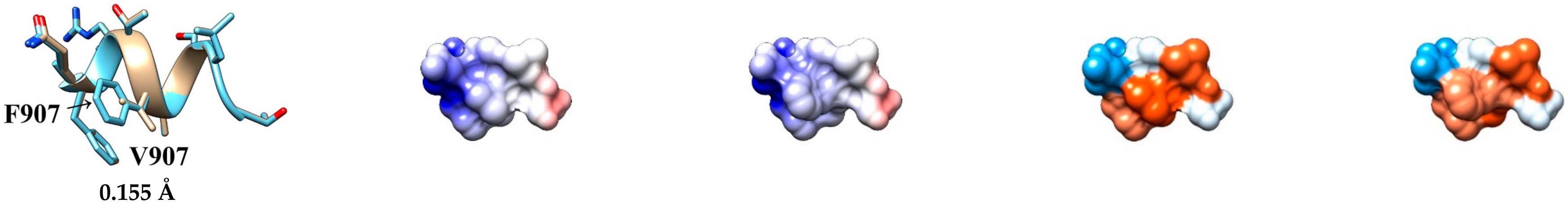

V907F

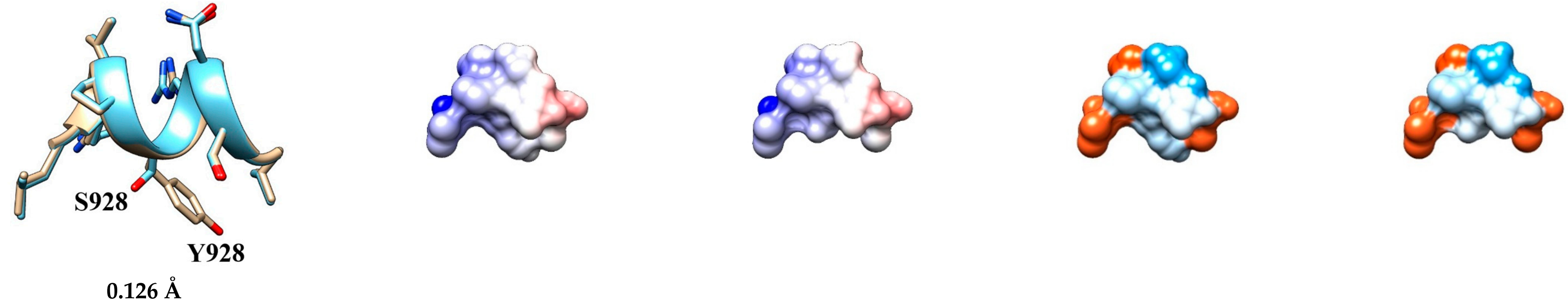

Y928S

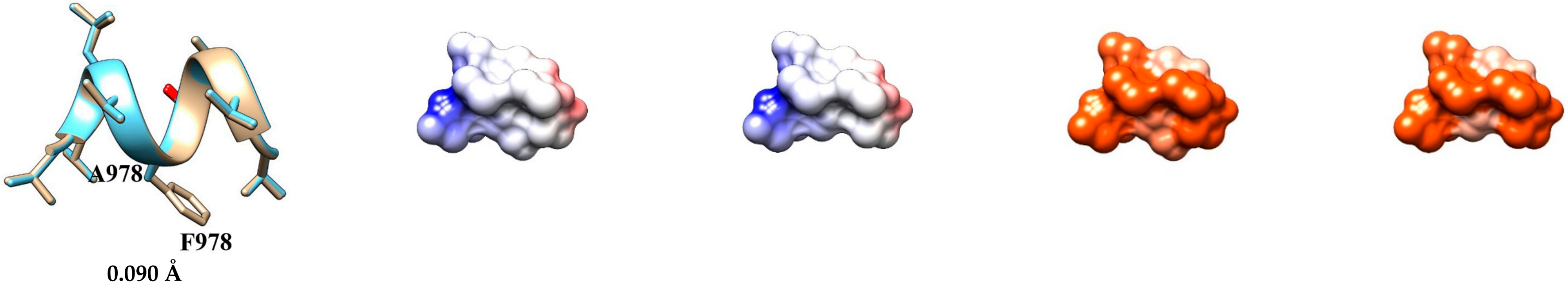

F978A

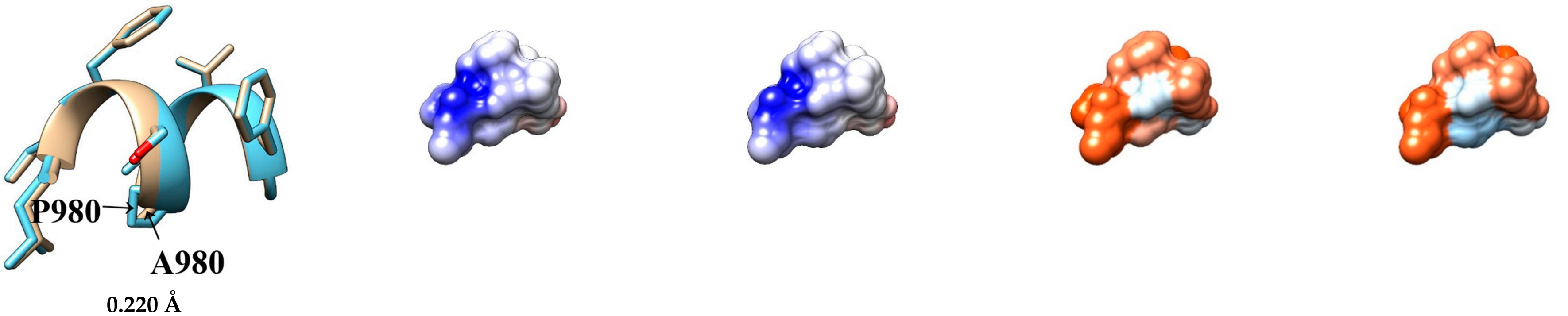

A980P

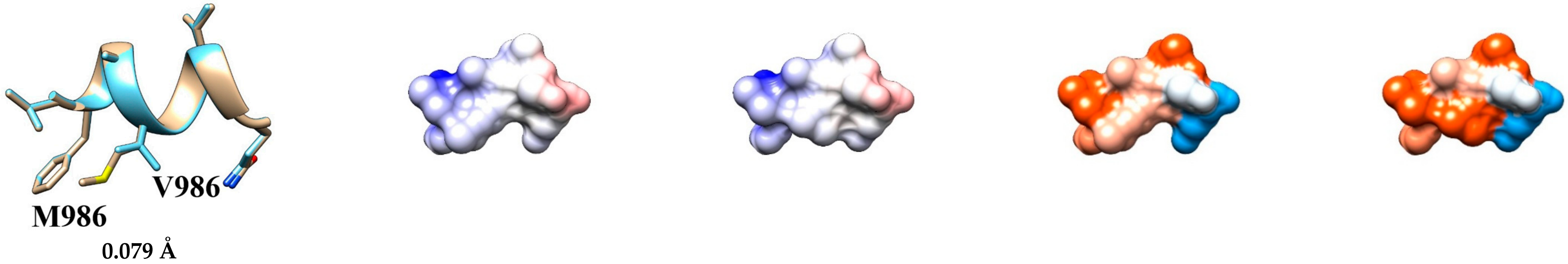

M986V

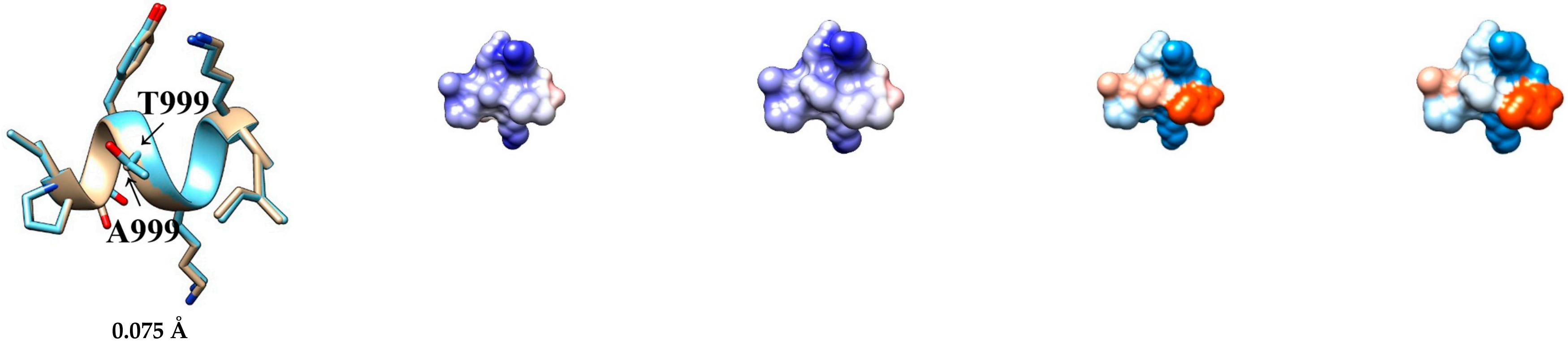

A999T

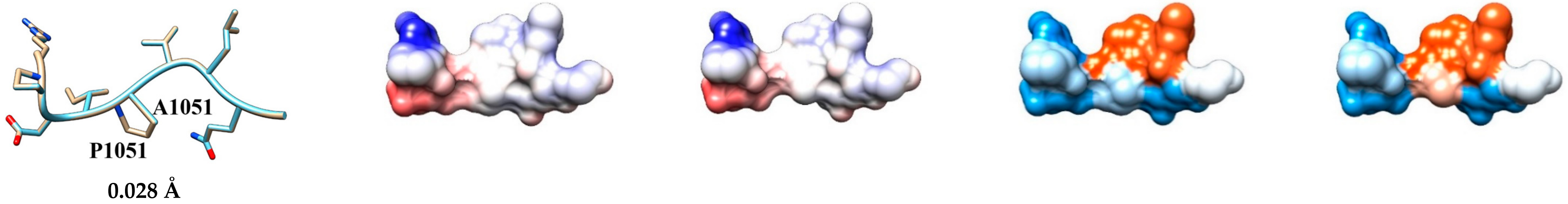

P1051A

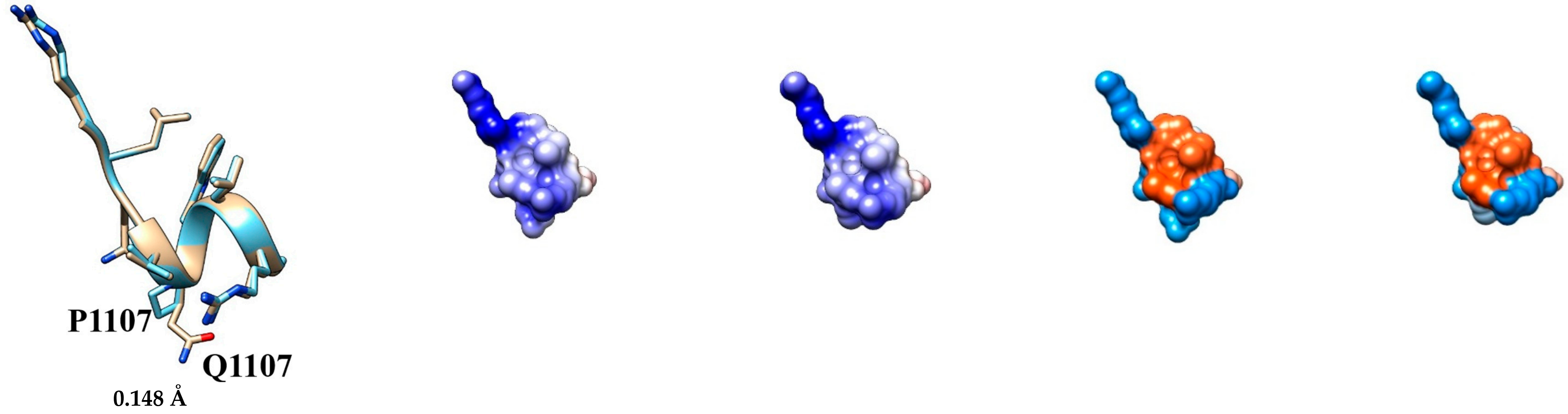

Q1107P

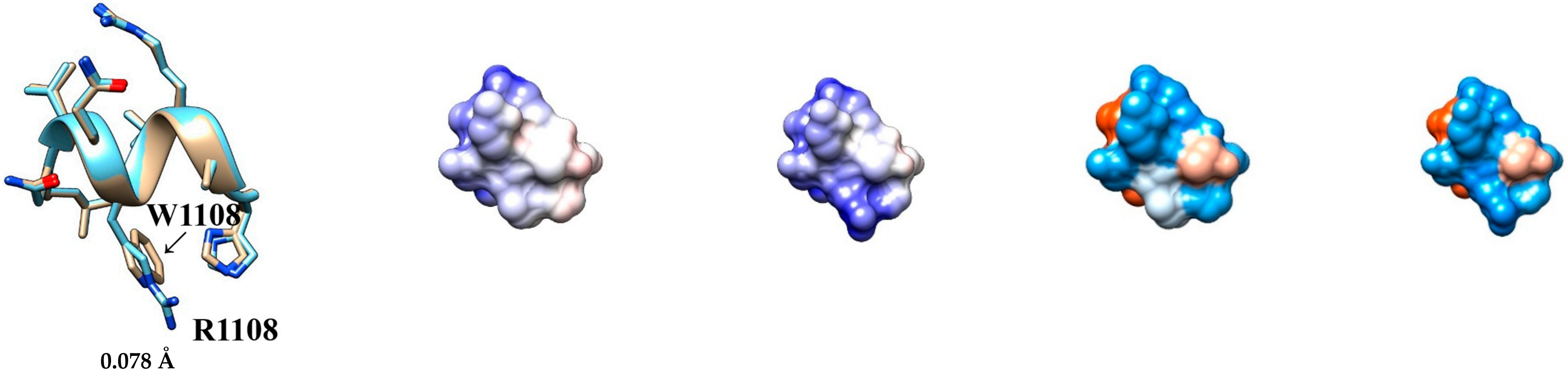

W1108R

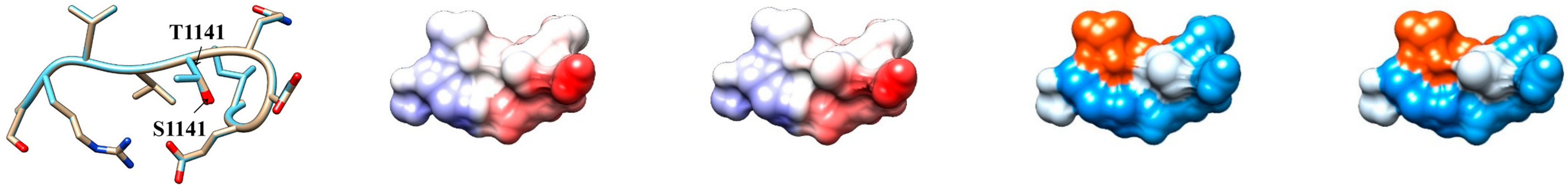

0.008 Å

S1141T

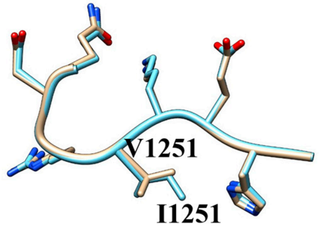

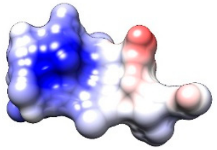

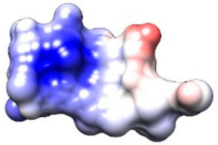

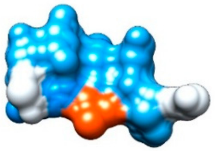

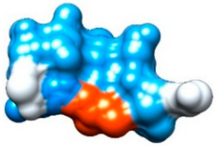

0.173 Å

V1251I

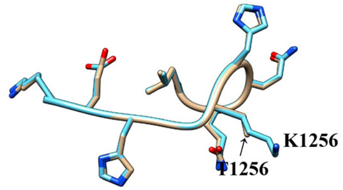

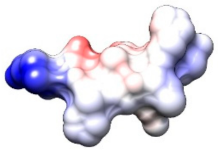

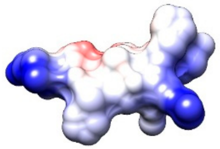

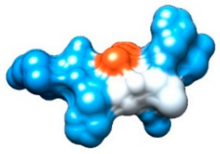

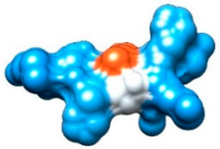

0.117 Å

T1256K
